# A bibliometric and scientific knowledge map study of the drug therapies for asthma-related study from 1982 to 2021

**DOI:** 10.3389/fphar.2022.916871

**Published:** 2022-10-03

**Authors:** Gao Zhen, Liu Yingying, Xu Weifang, Dong Jingcheng

**Affiliations:** ^1^ Department of Integrated Traditional Chinese and Western Medicine, Huashan Hospital Affiliated to Fudan University, Shanghai, China; ^2^ Institutes of Integrative Medicine, Fudan University, Shanghai, China; ^3^ Department of Retired Veteran Cadres, Putuo Hospital, Shanghai University of Traditional Chinese Medicine, Shanghai, China; ^4^ Shenzhen Hospital of Guangzhou University of Chinese Medicine, Shenzhen, China

**Keywords:** asthma, severe asthma, drug, bibliometric analysis, hotspots and frontiers

## Abstract

**Objective:** Asthma drug research has been increasing yearly, and its clinical application value has increasingly attracted attention. This study aimed to analyze the development status, research hotspots, research frontiers, and future development trends of the research works on drugs for patients with asthma, especially severe asthma.

**Methods:** Asthma drug-related articles published between 1982 and 2021 were retrieved from the Web of Science Core Collection (WOSCC) database, and only articles published in English were included. CiteSpace and VOSviewer software were utilized to conduct collaborative network analysis of countries/regions, institutions, keywords, and co-citation analysis of references.

**Results:** A total of 3,234 asthma drug-related eligible articles were included. The United States was in a leading position, and Karolinska Institute (Sweden) was the most active institution. The most prolific journal in this field was *Journal of Asthma*, and the most cited journal was *Journal of Allergy and Clinical Immunology*. Keyword co-occurrence studies suggested that the current hotspots and frontiers were as follows: ① asthma: fully revealing the potential of existing conventional asthma drugs, determining the best drug delivery system, and indicating the best combination. To continue to explore potential targets for severe asthma or other phenotypes. Inhaled glucocorticoids and budesonide are still one of the important aspects of current asthma drug research and ② severe asthma: the research and development of new drugs, especially monoclonal antibodies including omalizumab, mepolizumab, and benralizumab to improve asthma control and drug safety, have become a research hotspot in recent years, highlighting the importance of “target” selection.

**Conclusion:** This study demonstrates the global research hotspots and trends of the research works on drugs for patients with asthma/severe asthma. It can help scholars quickly understand the current status and hotspots of research in this field.

## 1 Introduction

Asthma is a heterogeneous disease and is mainly characterized by chronic airway inflammation. Its prevalence is increasing in several countries, especially among children, affecting 1%–18% of the population in different countries (Global Initiative for Asthma, 2020). The incidence rate of asthma among people who aged ≥20 years has reached 4.2% in China (Huang et al., 2019). Asthma has affected 30 million people in Western Europe, leading to impose a substantial burden on healthcare systems and economies (Fletcher et al., 2015). Current treatment strategies for asthma include inhaled corticosteroid (ICS), long-acting beta-agonist (LABA), and short-acting beta-agonist compounds, and in most cases they are effective, but sometimes patients showed inadequately controlled symptoms, repeated asthma exacerbations, or progressive decline in lung function. Acute exacerbations were commonly reported, in which 44% of respondents have used oral steroids for asthma in the previous 12 months, and 24% have referred to an emergency department, and 12% have been hospitalized (Price et al., 2014). The Global Initiative for Asthma (GINA) reports that approximately 3–10% of people with asthma have severe asthma (Global Initiative for Asthma, 2020). Despite implementation of an optimal management strategy, many patients with severe asthma are not able to achieve disease control (Roche et al., 2022). A meta-analysis showed that monoclonal antibodies (benralizumab, dupilumab, mepolizumab, omalizumab, and reslizumab) have demonstrated effectiveness in improving the clinical condition of severe uncontrolled asthma patients (Agache et al., 2020).

Symptom control and risk of adverse outcomes were included in asthma control (Global Initiative for Asthma, 2020). The proposal of this concept provides opportunities and challenges for asthma drug-related research works. However, the underlying mechanism of asthma, especially severe asthma, has still remained elusive, and asthma drug-related research works are also at a critical stage. In contrast to a systematic and scoping review, a bibliometric analysis refers to the qualitative and quantitative evaluation of a specific research field using mathematical and statistical methods to understand the knowledge structure and explore development trends (Donthu et al., 2021). The present study aimed to perform a comprehensive bibliometric analysis of asthma drug-related research publications from 1982 to 2021, with concentration on annual publications, subject categories, countries, journals, authors, keywords, and references. The results may provide hotspots and new trends in potential topics for future asthma drug-related research works.

## 2 Methods

### 2.1 Search strategy

A literature search was conducted to analyze all asthma drug-related articles using the Web of Science Core Collection (WOSCC) database from 1982 to 31 December 2021. The search strategy was performed as follows: Title (TI)=(asthma) and Topic Search (TS)=(drug). The search indices included the Science Citation Index Expanded (SCI-Expanded), Conference Proceedings Citation Index–Science (CPCI-S), and Current Chemical Reactions Expanded (CCR-Expanded). The search was completed on 12 April 2022. All publications were screened by two reviewers, and any discrepancies between reviewers (LYY and GZ) in the study selection were resolved *via* consultation with a third reviewer (DJC).

### 2.2 Data extraction and quality assessment

Data extraction and quality evaluation were performed independently. After searching in the WOSCC database, the number of publications and the total and average citations were recorded. All the retrieved publications met the following inclusion criteria: publication in English and the publication type is article. Those studies that were published in different form article type, such as review, conference abstract, editorial material, letter, bulletin, news item, etc., were excluded. In addition, the CiteSpace (Chen, 2006) (ver. 5.8.R1 and 6.1.R3) (Drexel University, Philadelphia, PA, United States) and VOSviewer (van Eck and Waltman, 2010) (ver. 1.6.17) (Leiden University, Leiden, Netherlands) software were utilized to analyze the overall structure of the network, the clustered network, the links between clusters, the key nodes or pivot points, and the pathways. A node in the map that represented the type of the study was analyzed, the size of the node indicated the number of publications, and links between the nodes represented relationships or collaborations, co-occurrence, or co-citations. Co-occurrence networks are a graphical representation of how frequently variables appear together. Systematic mapping outcomes include network and co-citation (or co-occurrence) clusters (Sabe et al., 2022). The interpretation of these clusters is augmented by CiteSpace’s automatic cluster labeling and summarization (Chen, 2006). The burstiness of the frequency of an entity over time indicates a specific duration when an abrupt change in the frequency takes place, thereby identifying emergent terms (Kleinberg, 2002). Units of measure were country, institutions, authors, journals, keywords, and references. For literature analysis, the time slice was 1 year, and the correlation strength was cosine. The threshold for each time slice selected was equal to 50.

## 3 Results

### 3.1 General data

From 1982 to 2021, 5,077 articles have been published. According to the classification of the WOS database, we only selected articles published in English. A total of 336 records were excluded in the first stage, of which 143, 69, 57, 24, 12, 9, 7, 4, 2, 2, 2, 1, 1, 1, 1, and 1 studies were published in French, German, Russian, Spanish, Italian, Polish, Portuguese, Turkish, Croatian, Japanese, Ukrainian, Chinese, Dutch, Icelandic, Norwegian, and Slovenian, respectively. In the second stage, 1,507 records were excluded, including 1,016, 202, 108, 75, 20, 28, and 58 studies that were published as review, conference abstract, editorial material, letter, bulletin, news item, and other forms, respectively. Finally, 3,234 eligible studies were included (Supplementary Figure S1). This study was designed in line with the Preferred Reporting Items for Systematic reviews and Meta-Analyses (PRISMA) checklist. Publications from 1982 to 31 December 2021 are listed in Supplementary Figure S2. The number of annual publications varied from three in 1982 to 183 in 2020. From 1982 to 1990, the first stage, the number of publications was not noticeable, with an average of (9.67 ± 4.36) articles per year. The second stage, from 1991 to 2017, is the stable period after growth, with an average of (92.81 ± 17.85) articles per year, and it rapidly increased in 2018. From 2018 to 2021, the third stage, the average number of published articles was (160.25 ± 15.65), accounting for 80.77% of the total publication. The majority of articles were published in 2020 (*n* = 183).

### 3.2 Distribution of countries and regions

**FIGURE 1:**
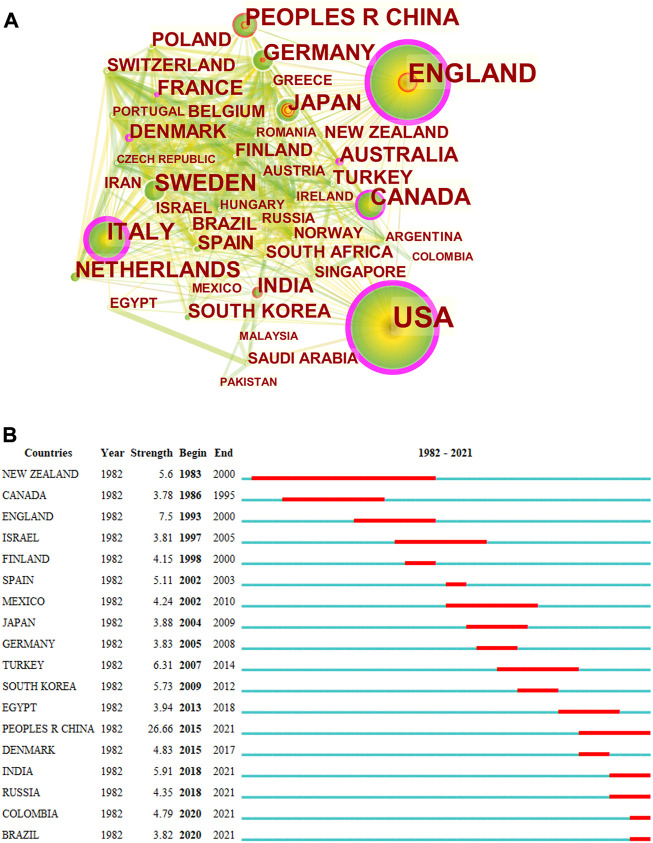
CiteSpace network map of countries/regions and top 18 countries with strongest citation bursts involved in asthma drug-related research. Notes: **(A)** CiteSpace network map of countries/regions involved in asthma drug-related research. The size of each node represents the number of publications of each country/region. The thickness of the links between nodes indicates the close degree of the cooperative relationship between countries/regions. **(B)** Top 18 countries with strongest citation bursts in asthma drug-related studies. The strongest citation burst means that a variable changes greatly in a short period. The blue line represents the base timeline, and the red part indicates the burst duration of each country.

Results of the intercountry/regional cooperation showed that 96 countries/regions have established partnerships, with 935 links among one other. Among the top 30 countries, there were 15 countries from Europe, eight countries from Asia, two countries from North America, two countries from Africa, two countries from Oceania, and one country from Latin America. The top five countries in terms of the number of published articles were the United States (*n* = 912, centrality = 0.37), the United Kingdom (*n* = 398, centrality = 0.28), Italy (*n* = 242, centrality = 0.29), China (*n* = 199, centrality = 0), and Canada (*n* = 199, centrality = 0.15) (Figure 1A). Countries/regions with the highest citation bursts are shown in Figure 1B. New Zealand presented the earliest and longest citation burst (5.6, 1983–2000), indicating that asthma drug-related research emerged in New Zealand from 1983–2000, followed by China (26.66, 2015–2021), India (5.91, 2018–2021), Russia (4.35, 2018–2021), Colombia (4.79, 2020–2021), and Brazil (3.82, 2020–2021), which had citation bursts until 2021. Among the top five countries in terms of publications, the annual number of publications of each country showed an increasing long-term trend, highlighting the importance of asthma drug-related research. Among them, the United States had the greatest number of publications, and the trend was relatively stable. China was the latest country that started asthma drug-related research among the top five countries by the number of publications, while the influence needs to be improved (Supplementary Figure S3).

### 3.3 Distribution of institutions

**FIGURE 2:**
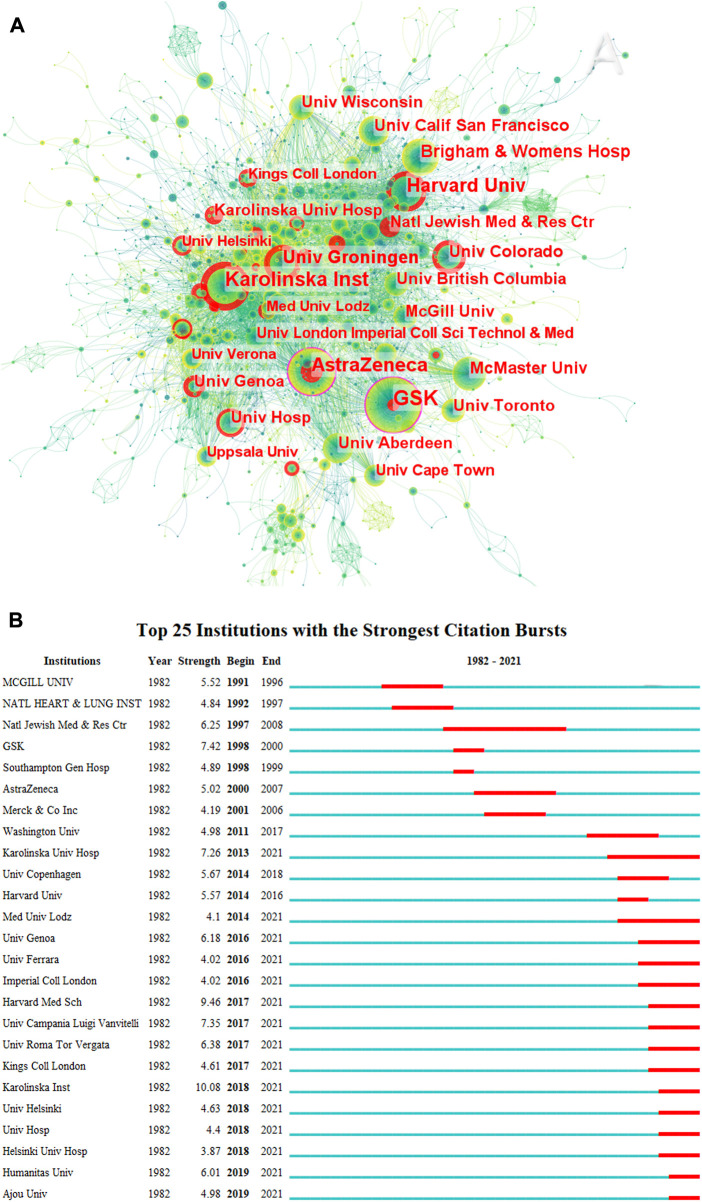
Co-occurrence map of institutions **(A)** and top 25 institutions with strongest citation bursts in asthma drug-related studies **(B)** (CiteSpace 6.1. R3).

According to the CiteSpace outputs, 3,166 different institutions were involved in this study. Figure 2A shows the distribution of institutions that published asthma drug-related articles between 1982 and 2021. GSK (United Kingdom) (*n* = 84, centrality = 0.15) is the institution with the highest number of asthma drug-related publications, followed by Karolinska Institute (Sweden) (*n* = 67, centrality = 0.04), AstraZeneca (United Kingdom) (*n* = 62, centrality = 0.11), Harvard University (United States) (*n* = 58, centrality = 0.07), and University of Groningen (Netherlands) (*n* = 42, centrality = 0.06). Supplementary Table S1 shows the top 20 institutions in terms of intermediary centrality and frequency, including six in the United States, four in the United Kingdom, four in Canada, and two in Sweden. The National Jewish Medical and Research Center presented the longest citation burst (6.25, 1997–2008). Karolinska University Hospital (7.26, 2013–2021), Medical University of Lodz (4.1, 2014–2021), University of Genoa (6.18, 2016–2021), University of Ferrara (4.02, 2016–2021), Imperial College London (4.02, 2016–2021), Harvard Medical School (9.46, 2017–2021), University of Campania Luigi Vanvitelli (7.35, 2017–2021), University of Roma Tor Vergata (6.38, 2017–2021), King’s College London (4.61, 2017–2021), Karolinska Institute (10.08, 2018–2021), University of Helsinki (4.63, 2018–2021), University Hospital (4.4, 2018–2021), Helsinki University Hospital (3.87, 2018–2021), Humanitas University (6.01, 2019–2021), and Ajou University (4.98, 2019–2021) had the bursts that continued to 2021 (Figure 2B). As shown in Supplementary Figure S4, it can be seen that Karolinska Institute is the earliest research institution that started asthma drug-related research and has continued to date and also performed well in the past 3 years.

### 3.4 Distribution of journals

**FIGURE 3:**
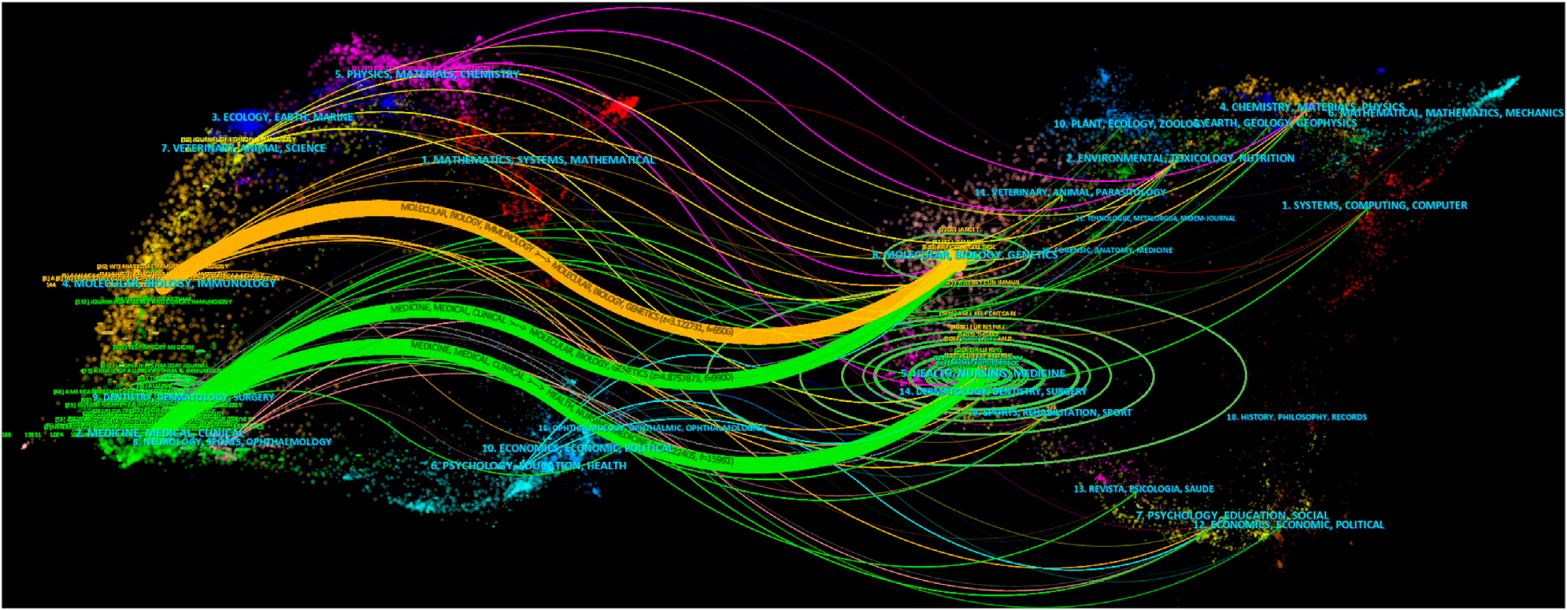
Dual-map overlay of journals on asthma drug-related studies. Notes: label top N journal: 50, font size: 8, a: 3, source circle size: 200, target circle size: 8, and snap to centroids (<radius): 0.

The top 20 cited journals and article-published journals are listed in Supplementary Table S2. The top three cited journals were Journal of Allergy and Clinical Immunology (2,199), European Respiratory Journal (1,709), and American Journal of Respiratory and Critical Care Medicine (1,686). The top three journals were Journal of Asthma (161), Journal of Allergy and Clinical Immunology (157), and Respiratory Medicine (103). The top 10 and three cited journals accounted for 43.58% and 17.17% of the total number of asthma-related journals, respectively. The top 10 and three journals accounted for 25.43% and 12.98% of the total number of asthma-related journals, respectively, and cited journals with the strongest citation bursts are shown in Supplementary Figure S5. British Medical Journal presented the earliest and longest citation burst (61.85, 1982–2004). From 2007 to date, the strongest citation bursts journal is Cochrane Database of Systemic Reviews (41.82, 2007–2021). Obviously, the following two stages were identified: the first stage lasted from 1982–2010, and the second stage spanned from 2011 to at least 2021. In the first stage, the journals were “British Medical Journal,” “Clinical Allergy,” “British Journal of Diseases of the Chest,” “European Journal of Respiratory Diseases,” “Annals of Allergy,” “American Review of Respiratory Disease,” “Archives of Disease in Childhood,” “Medical Journal of Australia,” “NIH Publications,” “Archives of Internal Medicine,” and “Archives of Pediatrics and Adolescent Medicine.” In the second stage, from 2011 to at least 2021, “Respiratory Research,” “Global Strategy for Asthma Management,” “Current Opinion in Allergy and Clinical Immunology,” “PLOS One,” “BMC Pulmonary Medicine,” “Current Allergy and Asthma Reports,” “Lancet Respiratory Medicine,” “Annals of the American Thoracic Society,” “Journal of Allergy and Clinical Immunology: In Practice,” “Journal of Asthma and Allergy,” “Allergology International,” and “Sci Rep-UK” were the most frequent journals. Figure 3 shows the distribution of journal topics and the citation relationship between journals. Additionally, there were three main references paths (one orange and two green) in it, suggesting that the articles published in “molecular/biology/genetics” journals were often cited by the articles published in “molecular/biology/immunology” and “medicine/medical/clinical.” The articles published in “health/nursing/medicine” journals were often cited by the articles published in “medicine/medical/clinical.”

### 3.5 Distribution of fields of asthma drug-related study

**FIGURE 4:**
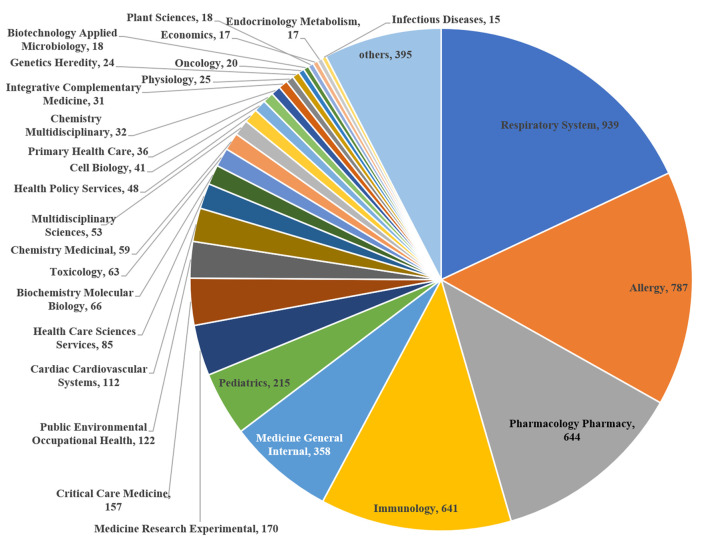
Research areas of asthma drug-related studies that were published from 1982 to 2021.

Respiratory system (*n* = 939, 18.03%), allergy (*n* = 787, 15.11%), pharmacology pharmacy (*n* = 644, 12.37%), immunology (*n* = 641, 12.31%), and general internal medicine (*n* = 358, 6.86%) totally accounted for 64.69% of the fields of study. This trend was followed by pediatrics (*n* = 215, 4.13%), experimental medicine research (*n* = 170, 3.26%), critical care medicine (*n* = 157, 3.01), public, environmental and occupational health (*n* = 122, 2.34%), and cardiac and cardiovascular systems (*n* = 112, 2.15) (Figure 4).

### 3.6 Analysis of co-occurring keywords and burst term

#### 3.6.1 Asthma

**FIGURE 5:**
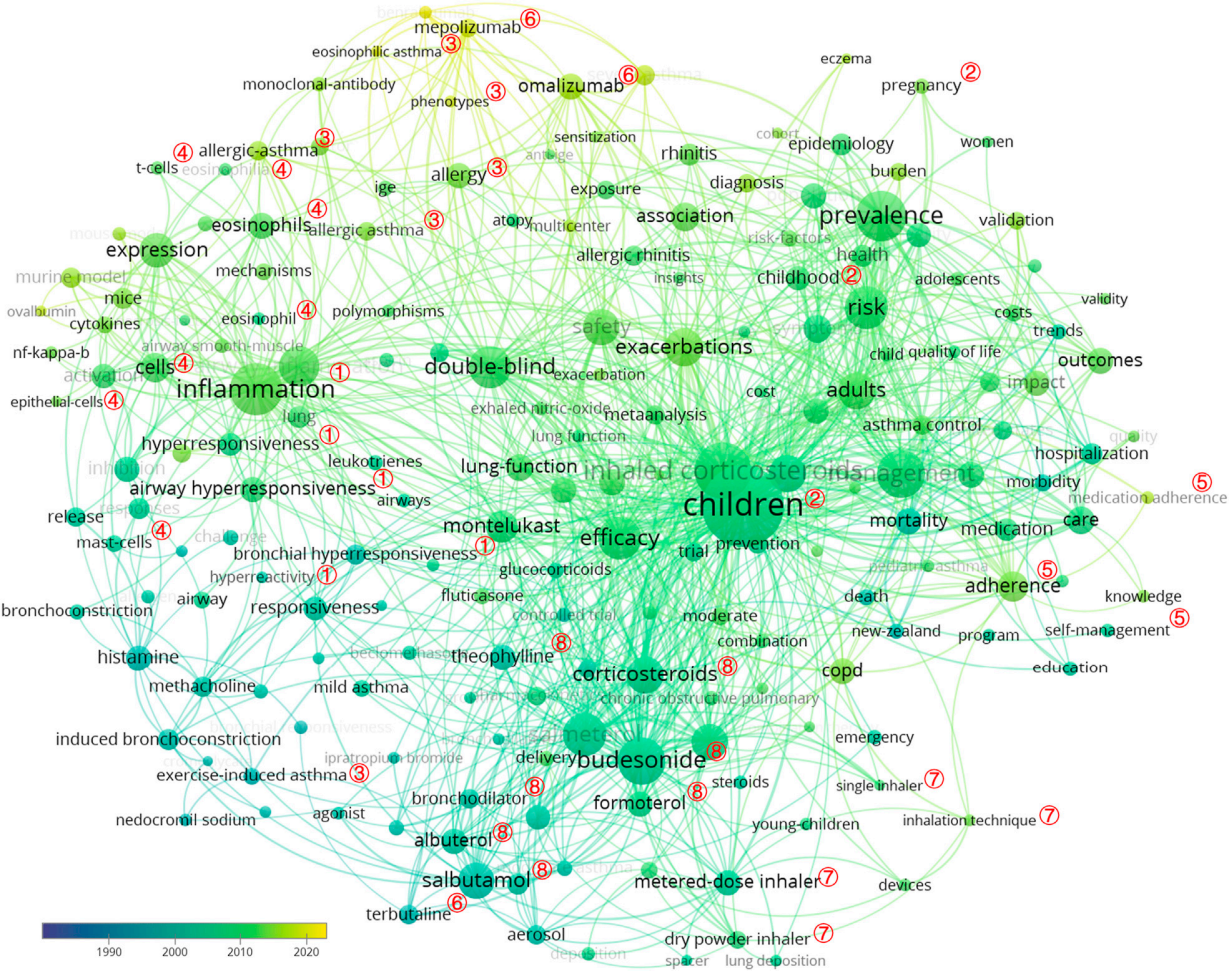
VOSviewer network map of co-occurrence analysis of all keywords of asthma drug-related studies. Notes: ① the basis of asthma: inflammation, airway hyperresponsiveness, bronchial hyperresponsiveness, hyperresponsiveness, and hyperreactivity; ② populations: children and pregnant women; ③ phenotypes: eosinophilic asthma, allergic asthma, and exercise-induced asthma; ④ cells: eosinophils, epithelial cells, mast cells, and T cells; ⑤ patient factors: adherence, medication adherence, self-management, and knowledge. ⑥ monoclonal antibody: omalizumab and mepolizumab; ⑦ drug delivery: metered-dose inhaler, dry powder inhaler, single inhaler, and inhalation technique; ⑧ conventional asthma drugs: budesonide, salbutamol, formoterol, theophylline, albuterol, terbutaline, bronchodilator, and corticosteroids.

Keywords represent the core of a scientific article. Thus, with analyzing keywords, we can track the knowledge evolution, hotspots, and future directions of research works (Zhang et al., 2022). A total of 425 keywords were obtained, and the top 30 meaningful keywords with the highest count are summarized in Table 1. After removing search-related terms and general words, including “asthma,” “drug,” “bronchial asthma,” “outcome,” “disease,” “expression,” “therapy,” and “association,” the top five keywords were children (*n* = 484, centrality = 0.06), inhaled corticosteroid (*n* = 310, centrality = 0.05), inflammation (*n* = 240, centrality = 0.03), prevalence (*n* = 229, centrality = 0.04), management (*n* = 210, centrality = 0.03), and budesonide (*n* = 207, centrality = 0.07). The top 200 keywords of asthma drug-related studies are visualized in Figure 5, and those keywords could be grouped by a thematic area as follows: ① the basis of asthma: inflammation, airway hyperresponsiveness, bronchial hyperresponsiveness, hyperresponsiveness, and hyperreactivity; ② populations with high incidence and need to clinicians’ attention: children and pregnant women; ③ phenotypes: eosinophilic asthma, allergic asthma, and exercise-induced asthma; ④ cells: eosinophils, epithelial cells, mast cells, and T cells; ⑤ patient-dependent factors: adherence, medication adherence, self-management, and knowledge; ⑥ monoclonal antibodies: omalizumab and mepolizumab; ⑦ drug delivery systems: metered-dose inhalers, dry powder inhalers, single inhalers, and inhalation techniques; and ⑧ conventional asthma drugs: budesonide, salbutamol, formoterol, theophylline, albuterol, terbutaline, bronchodilator, and corticosteroids. According to the frequency of occurrence, the top five asthma drugs were inhaled corticosteroids, budesonide, salbutamol, salmeterol, and corticosteroids.

**TABLE 1:** Top 30 keywords with the highest count in asthma drug-related studies.

Rank	Keyword	Count	Centrality	Rank	Keyword	Count	Centrality
1	Children	484	0.06	16	Safety	130	0.02
2	Inhaled corticosteroid	310	0.05	17	Childhood asthma	123	0.03
3	Inflammation	240	0.03	18	Fluticasone propionate	105	0.02
4	Prevalence	229	0.04	19	Montelukast	100	0.02
5	Management	210	0.03	20	Lung function	97	0.06
6	Budesonide	207	0.07	21	Eosinophil	94	0.06
7	Salbutamol	177	0.04	22	Adherence	94	0.02
8	Efficacy	175	0.02	23	Cell	92	0.04
9	Risk	170	0.02	24	Allergic asthma	86	0.01
10	Salmeterol	163	0.03	25	COPD	85	0.02
11	Double-blind	162	0.04	26	Quality of life	83	0.02
12	Exacerbation	161	0.04	27	Medication	83	0.03
13	Adult	150	0.01	28	Guideline	80	0.02
14	Corticosteroid	140	0.08	29	Omalizumab	78	0.01
15	Airway inflammation	139	0.03	30	Symptom	71	0.02

As shown in Table 2, a landscape generated using clusters of keywords presents the following seven blocks: #0 prevalence, #1 asthma, #2 inflammation, #3 safety, #4 theophylline, #5 drug, and #6 beta 2-adrenoceptor. As a result, asthma is an inflammatory disease, and inflammatory treatment for asthma is the focus of asthma drug-related research works.

**TABLE 2:** Co-occurrence map of asthma drug-related study keyword cluster analysis.

Cluster ID	Silhouette	Mean (year)	Label (LLR)
0	0.648	2001	Prevalence
1	0.585	1994	Asthma
2	0.554	2002	Inflammation
3	0.674	2002	Safety
4	0.786	1994	Theophylline
5	0.964	1990	Drug
6	0.978	2005	Beta 2-adrenoceptor

**FIGURE 6:**
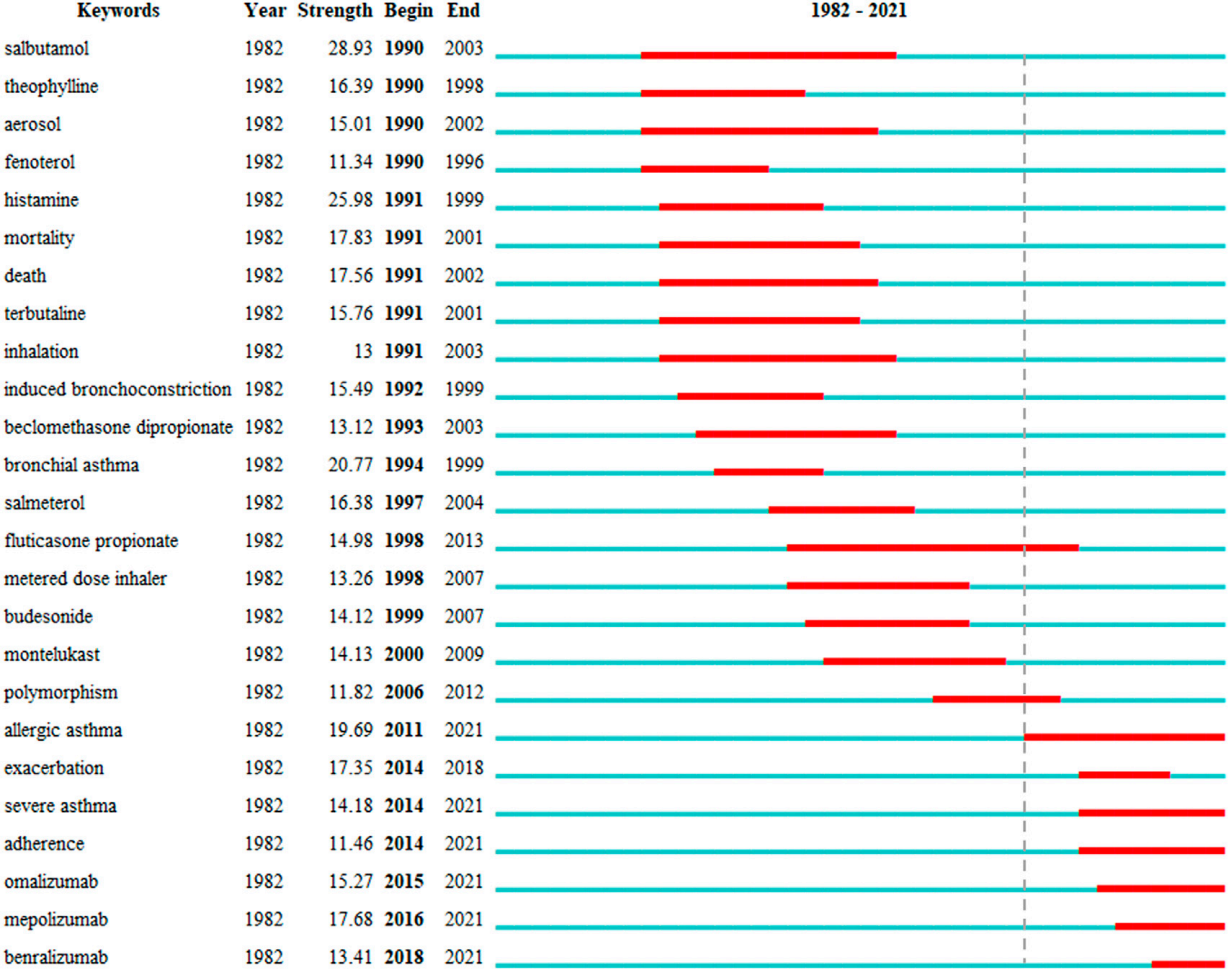
Top 25 keywords with strongest citation bursts in asthma drug-related studies.

Keywords with the strongest citation bursts in this field are presented in Figure 6. The earliest year in which the keywords appeared was indicated, including the time when the burst was started and ended. A red bar denotes the time when the keywords occur frequently, while the blue bar shows the time when the keywords occur infrequently. Overall, two stages were identified: the first stage lasted from 1990 to 2010, and the second stage spanned from 2011 to at least 2021. In the first stage, the keywords were “salbutamol,” “theophylline,” “aerosol,” “fenoterol,” “histamine,” “mortality,” “death,” “terbutaline,” “inhalation,” “induced bronchoconstriction,” “beclomethasone dipropionate,” “bronchial asthma,” “salmeterol,” “fluticasone propionate,” “metered-dose inhaler,” “budesonide,” “montelukast,” and “polymorphism.” In the second stage, from 2011 to at least 2021, “allergic asthma,” “exacerbation,” “severe asthma,” “adherence,” “omalizumab,” “mepolizumab,” and “benralizumab” were the most frequent keywords.

As shown in Table 3, there are 21 keywords of asthma drugs and nine keywords of asthma drug categories from 162 keywords with the strongest citation bursts in asthma drug-related studies. There were 13 asthma drugs, each with the number of publications ≥25, except for budesonide, and in most of the conventional asthma drugs, few studies were published after 2017. Among them, the most recent article on the study of nedocromil sodium in asthma therapy was published in 2000, and the most recent article on the study of fenoterol in asthma therapy was published in 2002. The monoclonal antibodies for asthma-related research works, sorted by time from far to near, were omalizumab, mepolizumab, and benralizumab, and related research articles were published every year in the past 4 years. The ICS in asthma-related research articles was published every year since 1996 to 2021 (Supplementary Figure S6).

**TABLE 3:** Keywords of asthma drugs with strongest citation bursts in asthma drug-related studies.

Rank	Keyword	Begin	End	Strength	Year
Drug					
1	Salbutamol	1990	2003	28.93	1982
2	Ipratropium bromide	1990	1993	5.60	1982
3	Fenoterol	1990	1996	11.34	1982
4	Terbutaline	1991	2001	15.76	1982
5	Methacholine	1991	2003	11.26	1982
6	Nedocromil sodium	1991	2000	11.25	1982
7	Cromolyn sodium	1992	1996	5.89	1982
8	Beclomethasone dipropionate	1993	2003	13.12	1982
9	Sodium cromoglycate	1993	2001	6.65	1982
10	Terfenadine	1993	1997	4.01	1982
11	Salmeterol	1997	2004	16.38	1982
12	Fluticasone propionate	1998	2013	14.98	1982
13	Budesonide	1999	2007	14.12	1982
14	Montelukast	2000	2009	14.13	1982
15	Zafirlukast	2000	2001	3.95	1982
16	Formoterol	2001	2008	7.87	1982
17	Beclomethasone	2001	2006	3.93	1982
18	Fluticasone	2004	2009	7.64	1982
19	Omalizumab	2015	2021	15.27	1982
20	Mepolizumab	2016	2021	17.68	1982
21	Benralizumab	2018	2021	13.41	1982
Drug category					
1	Glucocorticoid	1996	2003	5.01	1982
2	Theophylline	1990	1998	16.39	1982
3	Steroid	2001	2007	6.72	1982
4	Inhaled steroid	1997	2004	5.71	1982
5	Beta (2)-agonist	1997	2000	5.68	1982
6	Leukotriene receptor antagonist	1998	2008	11.03	1982
7	Bronchodilator	2008	2009	4.29	1982
8	Corticosteroid	2000	2003	4.27	1982
9	Monoclonal antibody	2017	2021	7.63	1982

#### 3.6.2 Severe asthma

**FIGURE 7:**
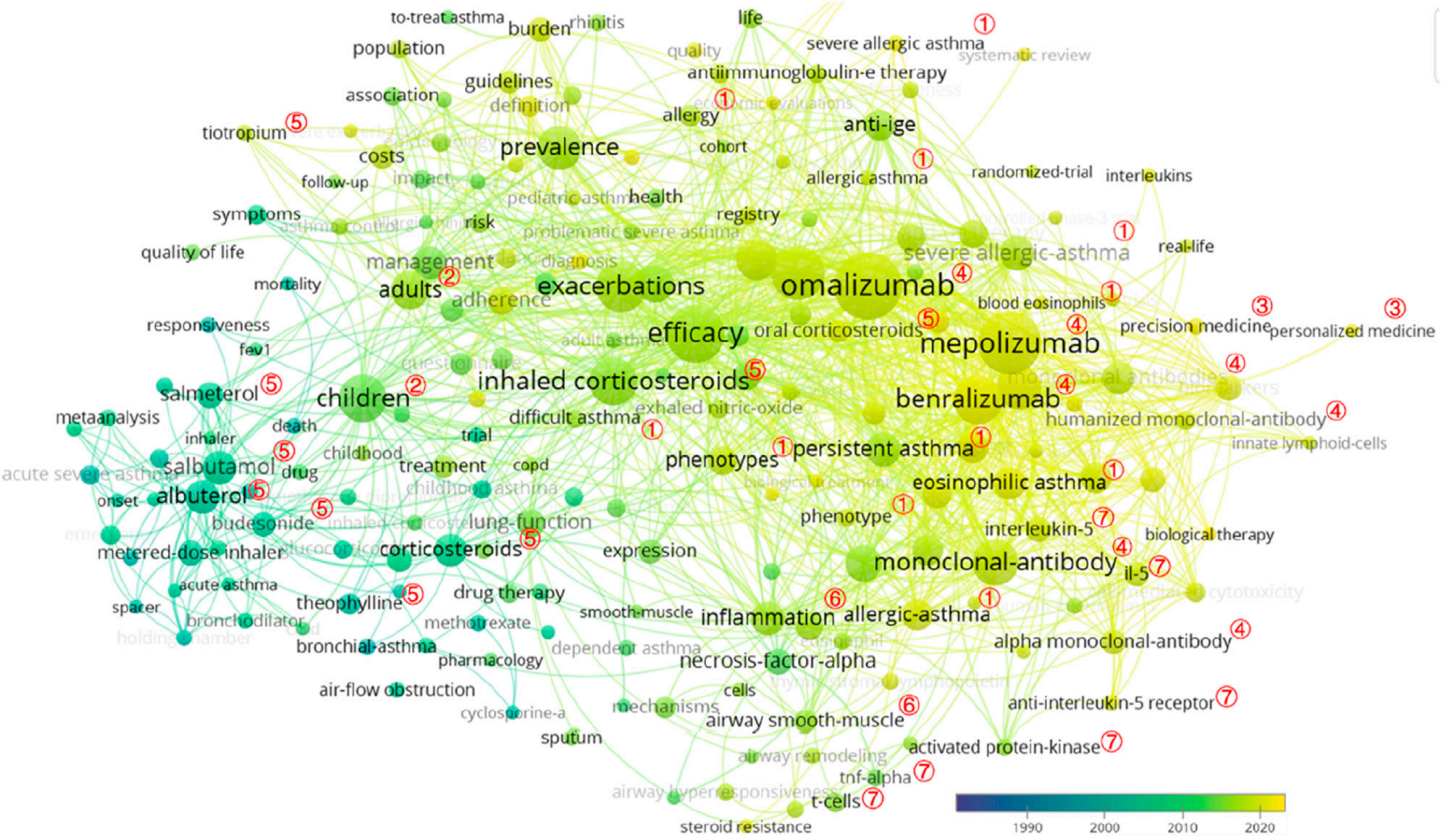
VOSviewer network map of co-occurrence analysis of all keywords in severe asthma. Notes: ① phenotypes: allergic asthma; eosinophilic asthma, persistent asthma, and difficult asthma; ② populations: children and adults; ③ precision treatment: precision medicine and personalized medicine; ④ monoclonal antibody: omalizumab, mepolizumab, and benralizumab; ⑤ conventional asthma drugs: budesonide, salbutamol, salmeterol, tiotropium, theophylline, and corticosteroids (inhaled corticosteroids and oral corticosteroids); ⑥ the basis of asthma: inflammation and airway smooth-muscle; ⑦ possible therapeutic targets: interleukin-5, interleukin-5 receptor, TNF-alpha, T cells, and activated protein kinase.

In total, 1,353 keywords were identified, and the top 30 meaningful keywords with the highest count are displayed in Table 4 and Figure 7. After removing search-related terms and general words, including “asthma,” “double-blind,” “severe asthma,” “therapy,” and “placebo,” the top five keywords were “omalizumab” (*n* = 71, centrality = 0.01), “mepolizumab” (*n* = 63, centrality = 0.01), “efficacy” (*n* = 55, centrality = 0.04), “exacerbation” (*n* = 49, centrality = 0.01), and “monoclonal antibody” (*n* = 49, centrality = 0.01). According to the frequency of occurrence, the top five severe asthma treatment drugs were “omalizumab,” “mepolizumab,” “benralizumab,” “ICS,” and “albuterol”.

**TABLE 4:** Top 30 keywords with the highest count in severe asthma drug-related studies.

Rank	Keyword	Count	Centrality	Rank	Keyword	Count	Centrality
1	Omalizumab	71	0.01	16	Severe allergic asthma	23	0.01
2	Mepolizumab	63	0.01	17	Persistent asthma	21	0.06
3	Efficacy	55	0.04	18	Eosinophil	20	0.06
4	Exacerbation	49	0.05	19	Albuterol	20	0.03
5	Monoclonal antibody	49	0.01	20	Allergic asthma	20	0.03
6	Safety	48	0.05	21	Airway inflammation	19	0.05
7	Benralizumab	39	0	22	Salbutamol	19	0.04
8	Children	37	0.16	23	Inflammation	19	0.02
9	Inhaled corticosteroid	35	0.03	24	Lung function	18	0.09
10	Prevalence	32	0.01	25	Corticosteroid	18	0.06
11	Quality of life	30	0.02	26	Eosinophilic asthma	18	0.02
12	Severe eosinophilic asthma	29	0	27	Receptor	18	0
13	Multicenter	28	0.03	28	Ige	17	0.04
14	Adult	26	0.03	29	Management	16	0.05
15	Phenotype	23	0.01	30	Cost	15	0.04

As shown in Table 5, a landscape generated using clusters of keywords presented the following 20 blocks, and the top 10 blocks were as follows: #0 severe allergic asthma, #1 omalizumab, #2 acute severe asthma, #3 severe asthma, #4 children, #5 IL-5, #6 nasal polyposis, #7 health economics, #8 association, and #9 emergency therapy.

**TABLE 5:** Co-occurrence map of severe asthma drug-related study keyword cluster analysis.

Cluster ID	Silhouette	Mean (year)	Label (LLR)
0	0.815	2012	Severe allergic asthma
1	0.758	2011	Omalizumab
2	0.791	2005	Acute severe asthma
3	0.768	2006	Severe asthma
4	0.719	2004	Children
5	0.745	2012	IL-5
6	0.861	2012	Nasal polyposis
7	0.912	2013	Health economics
8	0.943	2010	Association
9	0.933	2004	Emergency therapy
10	0.839	2007	Allergic bronchopulmonary aspergillosis
11	0.884	2005	Methotrexate
12	0.916	2009	Primary lysis/necrosis
13	0.925	2015	Hypoxia
14	0.91	2010	Cost-effectiveness
15	0.992	2001	Allergen
16	0.988	1999	Platelet-activating factor
17	0.997	2012	Flutiform
19	1	2005	Non-steroidal anti-inflammatory drugs (NSAID)
21	0.992	2020	Severe asthma research

**FIGURE 8:**
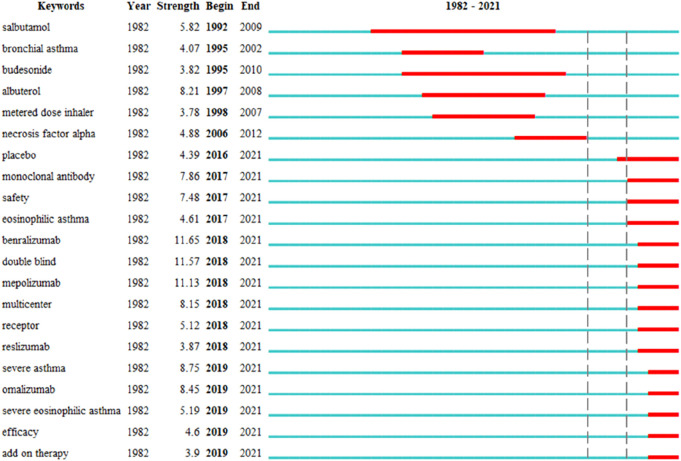
Top 21 keywords with strongest citation bursts in severe asthma drug-related studies.

As illustrated in Figure 8 and Table 6, two stages were identified: the first stage lasted from 1992 to 2012, and the second stage spanned from 2017 to at least 2021. In the first stage, the keywords were “salbutamol,” “bronchial asthma,” “budesonide,” “albuterol,” “metered-dose inhaler,” and “necrosis factor alpha.” In the second stage, from 2017 to at least 2021, “monoclonal antibody,” “safety,” “eosinophilic asthma,” “benralizumab,” “double-blind,” “mepolizumab,” “multicenter,” “receptor,” “reslizumab,” “severe asthma,” “omalizumab,” “severe eosinophilic asthma,” “efficacy,” and “add-on therapy” were the most frequent keywords. As presented in Supplementary Figure S7, the number of research articles on onomalizumab, mepolizumab, and benralizumab has grown steadily in recent years.

**TABLE 6:** Keywords of asthma drug with strongest citation bursts in severe asthma drug-related studies.

Year	Begin	End	Strength	Keyword
1982	1992	2009	5.82	Salbutamol
1982	1995	2010	3.82	Budesonide
1982	1997	2008	8.21	Albuterol
1982	2017	2021	7.86	Monoclonal antibody
1982	2018	2021	11.65	Benralizumab
1982	2018	2021	11.13	Mepolizumab
1982	2018	2021	3.87	Reslizumab
1982	2019	2021	8.45	Omalizumab

### 3.7 Analysis of cited and co-cited references

The citation count included the number of citations to a publication, and the co-citation count was defined as the frequency, in which two published articles were cited together by subsequently published articles (Small, 1973).

#### 3.7.1 Asthma

##### 3.7.1.1 Analysis of cited references

The 10 most frequently cited references from 1985 to 2016 are summarized in Table 7. Those references were cited more than 430 times, with the top two references that were cited more than 740 times each.

**TABLE 7:** Top 10 cited references in asthma drug-related studies.

Rank	Author/year	Cited reference	Source	Frequency
1	Wenzel et al. (2013)	Dupilumab in persistent asthma with elevated eosinophil levels	New England Journal of Medicine	857
2	Lötvall et al. (2011)	Asthma endotypes: a new approach to classification of disease entities within the asthma syndrome	Journal of Allergy and Clinical Immunology	749
3	Suissa et al. (2000)	Low-dose inhaled corticosteroids and prevention of death from asthma	New England Journal of Medicine	741
4	Rabe et al. (2004)	Worldwide severity and control of asthma in children and adults: The global asthma insights and reality surveys	Journal of Allergy and Clinical Immunology	686
5	Castro et al. (2015)	Reslizumab for inadequately controlled asthma with elevated blood eosinophil counts: results from two multicenter, parallel, double-blind, randomized, placebo-controlled, phase 3 trials	Lancet Respiratory Medicine	662
6	Bleecker et al. (2016)	Efficacy and safety of benralizumab for patients with severe asthma uncontrolled with high-dosage inhaled corticosteroids and long-acting beta 2-agonists (SIROCCO): a randomized, multicenter, placebo-controlled phase 3 trial	Lancet	635
7	Wenzel et al. (2016)	Dupilumab efficacy and safety in adults with uncontrolled persistent asthma despite the use of medium-to-high-dose inhaled corticosteroids along with a long-acting beta (2)-agonist: a randomized double-blind placebo-controlled pivotal phase 2 b dose-ranging trial	Lancet	508
8	Malmstrom et al. (1999)	Oral montelukast, inhaled beclomethasone, and placebo for chronic asthma—a randomized, controlled trial	Annals of Internal Medicine	469
9	Milgrom et al. (1996)	Non-compliance and treatment failure in children with asthma	Journal of Allergy and Clinical Immunology	466
10	Kraan et al. (1985)	Changes in bronchial hyper-reactivity induced by 4 weeks of treatment with anti-asthmatic drugs in patients with allergic asthma: a comparison between budesonide and terbutaline	Journal of Allergy and Clinical Immunology	431

##### 3.7.1.2 Analysis of co-cited references

**FIGURE 9:**
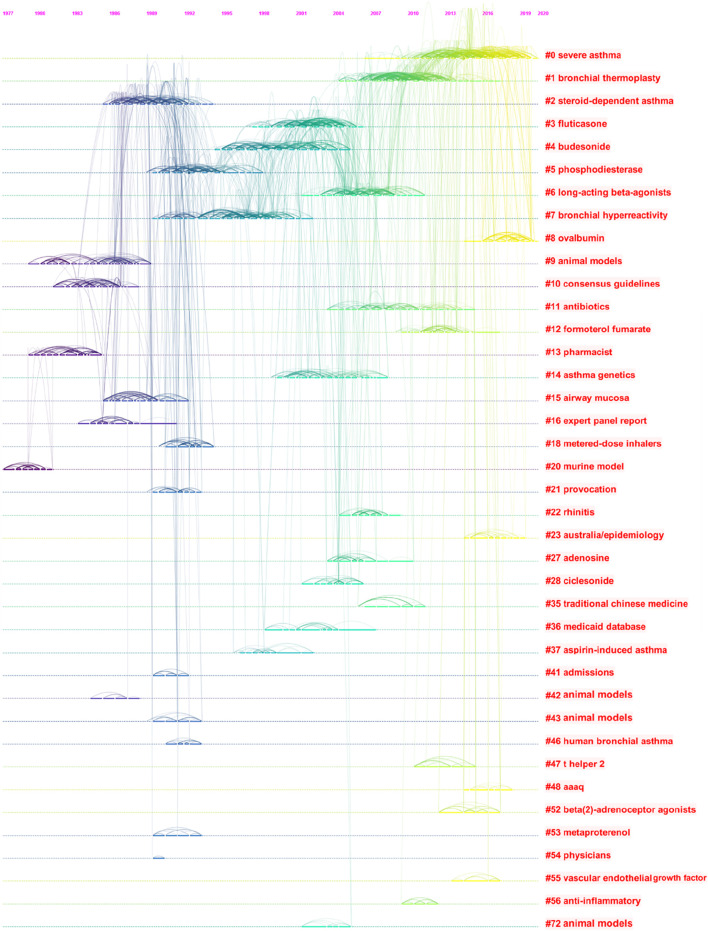
Timeline view for clusters of asthma drug-related study co-cited references.

CiteSpace could potentially partition the co-citation network into clusters, displaying firmly related references in one cluster and loosely connected references in another. Words from the titles of the cited articles inside the cluster were used to designate each cluster. We identified 32 different clusters in the network of co-cited references, with significant modularity and silhouette scores, indicating highly credible clusters (Q = 0.8744, S = 0.9287). The top 22 clusters are shown in Table 8 and Figure 9, including #0 severe asthma, #1 bronchial thermoplasty, #2 steroid-dependent asthma, #3 fluticasone, #4 budesonide, #5 phosphodiesterase, #6 long-acting beta-agonists, #7 bronchial hyperreactivity, #8 ovalbumin, #9 animal models, #11 antibiotics, #12 formoterol fumarate, #14 asthma genetics, #15 airway mucosa, #18 metered-dose inhalers, #21 provocation, #22 rhinitis, #23 Australia/epidemiology, #27 adenosine, #28 ciclesonide, #35 traditional Chinese medicine, and #36 Medicaid database.

**TABLE 8:** Co-occurrence map of asthma drug-related studies co-cited reference cluster analysis.

Cluster ID	Silhouette	Mean (year)	Label (LLR)
0	0.865	2015	Severe asthma
1	0.918	2009	Bronchial thermoplasty
2	0.814	1989	Steroid-dependent asthma
3	0.877	2002	Fluticasone
4	0.919	1998	Budesonide
5	0.938	1992	Phosphodiesterase
6	0.903	2006	Long-acting beta-agonists
7	0.943	1996	Bronchial hyperreactivity
8	0.964	2018	Ovalbumin
9	0.993	1984	Animal models
11	0.978	2009	Antibiotics
12	0.966	2012	Formoterol fumarate
14	0.967	2003	Asthma genetics
15	0.991	1988	Airway mucosa
18	0.999	1992	Metered-dose inhalers
21	0.997	1991	Provocation
22	0.996	2006	Rhinitis
23	0.994	2016	Australia/epidemiology
27	0.991	2005	Adenosine
28	0.997	2003	Ciclesonide
35	0.996	2008	Traditional Chinese medicine
36	0.996	2002	Medicaid database

#### 3.7.2 Severe asthma

##### 3.7.2.1 Analysis of cited references

The 10 most frequently cited references from 1992 to 2016 are summarized in Table 9. Those references were cited more than 183 times, with the top two references that were cited more than 370 times each.

**TABLE 9:** Top 10 cited references in severe asthma drug-related studies.

Rank	Author/year	Cited reference	Source	Frequency
1	Bleecker et al. (2016)	Efficacy and safety of benralizumab for patients with severe asthma uncontrolled with high-dosage inhaled corticosteroids and long-acting β2-agonists (SIROCCO): a randomized, multicenter, placebo-controlled phase 3 trial	Lancet	633
2	Salpeter et al. (2006)	Meta-analysis: effect of long-acting beta-agonists on severe asthma exacerbations and asthma-related deaths	Annals of Internal Medicine	371
3	Hekking et al. (2015)	Prevalence of severe refractory asthma	Journal of Allergy and Clinical Immunology	341
4	Alexander et al. (1992)	Trial of cyclosporin in corticosteroid-dependent chronic severe asthma	Lancet	329
5	Holgate and Polosa (2006)	Mechanisms, diagnosis, and management of severe asthma in adults	Lancet	278
6	Hanania et al. (2015)	Lebrikizumab in moderate-to-severe asthma: pooled data from two randomized placebo-controlled studies	Thorax	269
7	Brightling et al. (2015)	Efficacy and safety of tralokinumab in patients with severe uncontrolled asthma: a randomized, double-blind, placebo-controlled phase 2 b trial	Lancet Respiratory Medicine	253
8	Engelkes et al. (2015)	Medication adherence and the risk of severe asthma exacerbations: a systematic review	European Respiratory Journal	220
9	Wenzel (2005)	Severe asthma in adults	American Journal of Respiratory and Critical Care Medicine	217
10	McFadden (2003)	Acute severe asthma	American Journal of Respiratory and Critical Care Medicine	183

##### 3.7.2.2 Analysis of co-cited references for severe asthma

**FIGURE 10:**
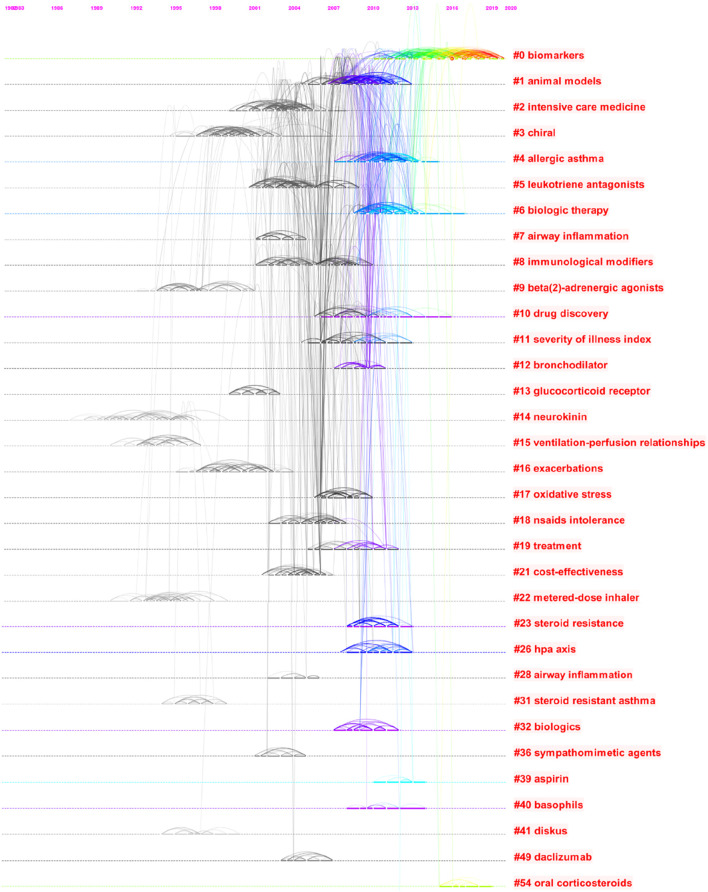
Timeline view for clusters of severe asthma drug-related study co-cited references.

The top 30 clusters are displayed in Table 10 and Figure 10, including #0 biomarkers, #1 animal models, #2 intensive care medicine, #3 chiral, #4 allergic asthma, #5 leukotriene antagonists, #6 biologic therapy, #7 airway inflammation, #8 immunological modifiers, #9 beta (2)-adrenergic agonists, #10 drug discovery, #11 severity of illness index, #12 bronchodilators, #15 ventilation–perfusion relationships, #16 exacerbations, #17 oxidative stress, #18 non-steroidal anti-inflammatory drug (NSAID) intolerance, #19 treatment, #21 cost-effectiveness, #22 metered-dose inhalers, #23 steroid resistance, #26 hypothalamic-pituitary-adrenal (HPA) axis, #31 steroid resistant asthma, #32 biologics, #36 sympathomimetic agents, #39 aspirin, #40 basophils, #41 diskus, #49 daclizumab, and #54 oral corticosteroids. Four different major trends of asthma drug-related research works were found. These included two clusters related to the severe asthma phenotypes (#4 and #31), seven clusters related to the types of asthma drugs (#5, #6, #8, #9, #12, #36, and #54), one cluster related to establishment of asthma models (#1), and one cluster related to monoclonal antibody (#49).

**TABLE 10:** Co-occurrence map of severe asthma drug-related study co-cited references cluster analysis.

ClusterID	Silhouette	Mean (year)	Label (LLR)
0	0.938	2016	Biomarkers
1	0.856	2009	Animal models
2	0.957	2003	Intensive care medicine
3	0.942	1999	Chiral
4	0.958	2011	Allergic asthma
5	0.9	2004	Leukotriene antagonists
6	0.905	2011	Biologic therapy
7	1	2002	Airway inflammation
8	0.953	2004	Immunological modifiers
9	0.992	1996	Beta (2)-adrenergic agonists
10	0.975	2009	Drug discovery
11	0.973	2008	Severity of illness index
12	0.975	2009	Bronchodilator
15	1	1993	Ventilation–perfusion relationships
16	1	1999	Exacerbations
17	0.967	2008	Oxidative stress
18	0.954	2005	NSAID intolerance
19	0.996	2009	Treatment
21	0.955	2004	Cost-effectiveness
22	0.986	1994	Metered-dose inhaler
23	0.995	2010	Steroid resistance
26	0.981	2010	HPA axis
31	1	1996	Steroid-resistant asthma
32	0.994	2009	Biologics
36	0.995	2003	Sympathomimetic agents
39	1	2012	Aspirin
40	1	2010	Basophils
41	1	1996	Diskus
49	0.999	2005	Daclizumab
54	0.998	2017	Oral corticosteroids

## 4 Discussion

### 4.1 General information

The number of publications related to asthma drugs is increasing year-by-year, especially the third stage in recent years. In the present study, the leading countries and regions, institutions, journals, authors, and research fields were presented, and the asthma drug-related research hotspots and trends were analyzed by using CiteSpace and VOSviewer software. Over the past 30 years, drug-related research works on asthma and severe asthma have been characterized by a phased growth. This feature may be related to how long it takes to identify new pathogenesis and drug targets in asthma.

#### 4.1.1 Countries and regions

The United States was the country with the highest number of publications, accompanied by dominancy of asthma drug-related research works and participating in more exchange and cooperation in asthma drug-related research works, followed by the United Kingdom and Italy. The number of publications was increasing year-by-year since 2019 in China and presented the most recent and the strongest citation burst. China was equal to the United States for the first place in asthma drug-related publications in 2021, while the citations and centrality of publications in China were significantly lower than those in the United States.

#### 4.1.2 Institutions

Karolinska Institute had the greatest number of publications. Karolinska Institute presented the most recent and the strongest citation burst. This visualization map enabled us to assess the influence and burstiness of the most important institutions with major hotspots. The National Jewish Medical and Research Center presented the longest citation burst from 1997 to 2008.

#### 4.1.3 Journals

The top three cited journals were Journal of Allergy and Clinical Immunology, European Respiratory Journal, and American Journal of Respiratory and Critical Care Medicine. The top three journals that published the most asthma drug-related research works were *Journal of Asthma*, *Journal of Allergy and Clinical Immunology*, and *Respiratory Medicine*, indicating that the top journals in the field of respiratory diseases were further concentrated on asthma-related medication. *Cochrane Database of Systemic Reviews*, as the world’s most important and influential database of evidence on clinical practice and medical decision-making, continued to date from 2007 and with the strongest citation bursts, suggesting that the role of evidence-based medicine in the study of asthma drugs has noticeably attracted scholars’ attention. Moreover, over the past decade, the influence of open-access (OA) journals has been highlighted.

### 4.2 The analysis of hotspots and trends

High-frequency keywords mainly reflect current issues and frontier trends in a specific research field; thus, the statistical analysis of keywords can quickly and effectively clarify the research hotspots and trends in this field (Yang et al., 2022). Research hotspots can be identified by keyword co-occurrence networks and cluster analysis.

#### 4.2.1 Asthma

The keywords could be grouped by the thematic area, and for asthma drug-related research works, the following keywords were subsequently used:

a. Conventional asthma drugs: ICSs, budesonide, salbutamol, salmeterol, and montelukast.
b. Monoclonal antibody: omalizumab.
c. The basis of asthma: inflammation and airway inflammation.
d. Pulmonary drug delivery: metered-dose inhalers, dry powder inhalers, single inhalers, and inhalation techniques.
e. Phenotypes: allergic asthma, eosinophilic asthma, and exercise-induced asthma.
f. Children and pregnant women.
g. Advise to asthma patients: adherence.

The results indicated that corticosteroids (budesonide and fluticasone propionate) and beta 2-agonists (salmeterol and salbutamol) played an important role in the management of asthma patients. The research hotspots and frontiers of asthma drug-related research works are reflected in the following three aspects: first, fully revealing the potential of existing conventional asthma drugs, determining the best drug delivery system, and indicating the best combination. Improving patient adherence and the correct use of inhalation devices can ensure effectiveness of drugs. Second, the factors that affect the efficacy of inhaled preparations are complex, such as inhalation method, hand–mouth coordination, and patients’ preference for the type of device, all of which may influence the efficacy of drugs.

Children, pregnant women, and other groups have special physiological states; thus, it is essential to develop a more easy-to-operate and efficient pulmonary drug delivery system. Third, it is suggested to continue to explore potential targets for severe asthma or other phenotypes.

Strong citation bursts of keywords were used to identify and analyze the research hotspots, frontiers, and emerging trends over time (Lyu et al., 2022). The results of asthma drug-related research works showed that, in the first stage (1990–2010), the hotspots and frontiers of asthma drug-related research works were conventional asthma drugs, including “salbutamol,” “theophylline,” “fenoterol,” “terbutaline,” “beclomethasone dipropionate,” “salmeterol,” “fluticasone propionate,” “budesonide,” and “montelukast.” In the second stage (2011 to at least 2021), the citation bursts of keywords, such as “allergic asthma,” “severe asthma,” “adherence,” “omalizumab,” “mepolizumab,” and “benralizumab,” continued to 2021 and is still ongoing, demonstrating that these directions have a great potential. Moreover, “allergic asthma” presented the most recent and the strongest citation burst. We selected asthma drugs in all 162 keywords with the strongest citation bursts and found that “fluticasone propionate” presented the longest citation burst. In addition, “leukotriene receptor antagonist (LTRA)” presented the longest citation burst in the drug category. The LTRA can effectively inhibit cysteinyl leukotrienes, reduce airway inflammation, and facilitate the downregulation of ICS doses (Global Initiative for Asthma, 2020). It is an alternative first-line drug for asthma control in children (Asthma group of Chinese thoracic society, 2020). Since 2015, monoclonal antibodies, such as omalizumab, mepolizumab, and benralizumab, appear at the research hotspots and frontiers of asthma drug research. Subsequent studies on severe asthma have shown that this trend is closely related to the phenotype of severe asthma.

#### 4.2.2 Severe asthma

For severe asthma drug-related research works, the following keywords were subsequently used:

a. Monoclonal antibodies: omalizumab, mepolizumab, and benralizumab.
b. Conventional asthma drugs: ICSs, salbutamol, albuterol, and corticosteroids.
c. Phenotypes: severe eosinophilic asthma, severe allergic asthma, persistent asthma, allergic asthma, and eosinophilic asthma.
d. Children need special attention.
e. The basis of asthma: inflammation and airway inflammation.

It has been indicated that eosinophils play a central role in the inflammation of asthma and are the target of new biological treatments for patients with severe asthma (Katsoulis et al., 2022).

The research hotspots and frontiers of severe asthma drug-related research works are reflected in the following three aspects: first, how to improve the clinical efficacy of severe asthma by changing the dose and combination and route of administration of conventional asthma drugs; second, defining the phenotypic characteristics of severe asthma and their therapeutic targets; and third, to develop new targeted drugs including monoclonal antibodies to improve asthma control and drug safety.

In addition to childhood asthma and pregnant women with asthma, the management of difficult-to-control asthma in adults remains an important challenge. Analysis of word frequency suggested that children are key population for asthma drug-related research works, and controlling inflammation has been found as a hotspot in asthma drug-related research works. Studies showed that asthma is the most common chronic disease of childhood and the most common respiratory condition in Ireland (Kabir et al., 2011). Sichuan, Southwest China, shows that the prevalence and years lived with disability of asthma children under the age of 5 years increased over the past 30 years, and male children are the key population of increasing asthma disease burden and deserve more attention (Luo et al., 2022).

As for severe asthma, the research and development of new drugs, especially monoclonal antibodies, have become a research hotspot in recent years, highlighting the importance of “target” selection. In the management of severe asthma, some essential areas, such as the improvement of pediatric asthma, severe allergic asthma, and outcomes, were identified as a hot topic. The concentration of severe asthma drug-related research works is different from that of general asthma to some extent, and for the severe asthma drug-related research works, from the beginning, it is basically the same as that of mild-to-moderate asthma, while the dosage, combination of drugs, and drug delivery system may be different, to the development of monoclonal antibodies for severe asthma; during this process, the identification of asthma targets and their functions plays an important role, providing a reference for the study of other phenotypes of asthma.

The results of severe asthma drug-related research works showed that there was a 4-year gap between Phase 1 and Phase 2, and the hotspots and frontiers of severe asthma drug-related research works in the first stage were conventional asthma drugs, including “salbutamol,” “budesonide,” and “albuterol.” In the second stage, the citation bursts of keywords, such as “monoclonal antibody,” “safety,” “eosinophilic asthma,” “benralizumab,” “double-blind,” “mepolizumab,” “multicenter,” “receptor,” “reslizumab,” “severe asthma,” “omalizumab,” “severe eosinophilic asthma,” “efficacy,” and “add-on therapy,” continued to 2021 and is still ongoing, demonstrating that these directions have a great potential. The research hotspots and frontiers of asthma concentrate on severe asthma, while the research hotspots and frontiers of drugs for severe asthma concentrate on monoclonal antibodies, including omalizumab, mepolizumab, and benralizumab.

#### 4.2.3 Monoclonal antibodies

The study showed that 83.8% severe asthma cases were identified as most likely (grade 3) to have an eosinophilic phenotype (Heaney et al., 2021). The phenotyping of severe asthma allows the precise use of biologics. Studies showed that, omalizumab—a monoclonal antibody which targets immunoglobulin E—represents the first available humanized monoclonal anti-IgE for use in pediatric severe allergic asthma (approved for use in children ≥ 6  years of age), with an established efficacy and safety profile (Licari et al., 2019). Mepolizumab—a monoclonal antibody against interleukin-5— is an effective and well-tolerated treatment that reduces the risk of asthma exacerbations in patients with severe eosinophilic asthma (Pavord et al., 2012) and also be useful in an emergency to treat steroid-refractory eosinophilic acute severe asthma (Barbarot et al., 2022). Phenotype-directed therapy with mepolizumab in urban children (aged 6–17 years) with exacerbation-prone eosinophilic asthma reduced the number of exacerbations (Jackson et al., 2022). In patients with persistent, moderate-to-severe asthma and elevated eosinophil levels who used inhaled glucocorticoids and LABAs, dupilumab therapy, as compared with placebo, was associated with fewer asthma exacerbations when LABAs and inhaled glucocorticoids were withdrawn, with improved lung function and reduced levels of Th2-associated inflammatory markers (Wenzel et al., 2013). Dupilumab binds to the alpha subunit of the IL-4 receptor, inhibiting its effects. Benralizumab—an anti-interleukin-5 (IL-5) receptor *α* monoclonal antibody—that treats patients with severe eosinophilic asthma showed a clinical efficacy of approximately 60% based on the Global Evaluation of Treatment Effectiveness score and may significantly improve the FEV1 in some patients with previous mepolizumab treatment (Numata et al., 2020). The results also confirmed that ICSs remain the most effective anti-inflammatory drugs for asthma (Diamant et al., 2007), while additional attention should be paid to the adverse reactions caused by excessive use of ICSs (Zhang et al., 2011).

### 4.3 Knowledge base

The most frequently cited references were examined to determine the key knowledge base in asthma (Lyu et al., 2022), the co-cited references disclosed how often two articles were cited together by other articles, and this can be seen as the basis of knowledge in a specialized area (Chen, 2006).

#### 4.3.1 Asthma

In the top 10 cited articles, some vital information was obtained as follows:

a. One conventional asthma therapy: low-dose ICSs, the regular use of low-dose ICSs is associated with a decreased risk of death from asthma.
b. Three conventional asthma drugs: oral montelukast and inhaled beclomethasone. Beclomethasone has a larger mean effect than montelukast, and both drugs are clinically beneficial for patients with chronic asthma. Budesonide: it can improve bronchial hyperreactivity.
c. Three monoclonal antibodies: dupilumab, it is associated with increased lung function and reduced severe exacerbations in patients with uncontrolled persistent asthma. Reslizumab: it can be used for patients with asthma and elevated blood eosinophil counts who are inadequately controlled by ICS-based therapy. Benralizumab: it can be used for patients with severe asthma and elevated eosinophils.
d. A new approach to classification of asthma: asthma endotypes.
e. One target population: children.
f. Advice to asthma patients: adherence, low rates of compliance with prescribed ICSs are associated with exacerbation of disease.

There were 22 major clusters in the co-citation network map of asthma drug-related research works, where “severe asthma” was the largest cluster, and eight different major themes of asthma drug-related research works were found, including two clusters related to the asthma phenotypes (“severe asthma” and “steroid-dependent asthma”), four clusters related to asthma drugs (“fluticasone,” “budesonide,” “formoterol fumarate,” and “ciclesonide”), two clusters related to types of asthma drugs (“long-acting beta-agonists” and “antibiotics”), two clusters related to establishment of asthma models (“ovalbumin” and “animal models”), one cluster related to traditional medicine (“traditional Chinese medicine”), one cluster related to non-drug therapy (“bronchial thermoplasty”), one cluster related to a promising target in the treatment of asthma (” phosphodiesterase”), and one cluster related to pulmonary delivery (“metered-dose inhalers”).

Those articles laid the foundation for further research into the structure and mechanism of asthma drugs and provided a theoretical basis for the study of asthma drug-related research works.

#### 4.3.2 Severe asthma

In the top 10 cited articles, the first highly co-cited article on severe asthma was “Efficacy and safety of benralizumab for patients with severe asthma uncontrolled with high-dosage inhaled corticosteroids and long-acting β2-agonists (SIROCCO): a randomized, multicenter, placebo-controlled phase 3 trial” (Bleecker et al., 2016). Among the nine articles, Salpeter SR found that long-acting beta-agonists have been shown to increase severe and life-threatening asthma exacerbations, as well as asthma-related deaths (Salpeter et al., 2006). The third article showed that severe refractory asthma accounted for 3.6% of adult asthma patients, indicating that there were 10.4 patients per 10,000 inhabitants (Hekking et al., 2015). In addition, there were three drugs for severe asthma (cyclosporin: the frequency of disease exacerbations requiring an increased prednisolone dose was reduced by 48% in patients treated with cyclosporin compared with placebo. Diurnal variation in the peak expiratory flow rate was reduced by a mean of 27.6% (Alexander et al., 1992). Lebrikizumab, a monoclonal antibody to IL-13: it reduced the rate of asthma exacerbations and increased FEV1, especially in the periostin-high asthma patients (Holgate and Polosa, 2006). Tralokinumab: it caused improvement in FEV1, with tralokinumab given every 2 weeks, and a possible treatment effect in a defined population of patients with severe uncontrolled asthma (Hanania et al., 2015). As for asthma patients, a good adherence was found to be associated with lower risks of severe asthma exacerbations (Brightling et al., 2015).

There were 30 major clusters in the co-citation network map of severe asthma drug-related research works, “biomarkers” would be the largest cluster, and four different major themes of severe asthma drug-related research works were found, including two clusters related to the severe asthma phenotypes (“allergic asthma” and “steroid resistant asthma”), seven clusters related to the types of asthma drugs (“leukotriene antagonists,” “biologic therapy,” “immunological modifiers,” “beta (2)-adrenergic agonists,” “bronchodilator,” “sympathomimetic agents,” and “oral corticosteroids”), one cluster related to establishment of asthma models (“animal models”), and one cluster related to monoclonal antibody (“daclizumab”). Asthma drug-related research works provided an early reference for development of severe asthma drug-related research works, while additional attention should be paid to severe asthma drug-related research works. Exploration of the underlying mechanisms of asthma is the key to develop therapeutic approaches, facilitating research works on asthma phenotype or subtype drugs including severe asthma, resulting in higher efficacy and safety. Good adherence and self-management are important aspects of this process to minimize the influence of asthma in real life, and a previous study showed that the benefits of daily regular administration of ICSs were diminished when adherence was low (50%) (Singh et al., 2022). Therefore, how to improve the adherence of asthma, especially mild-to-moderate asthma, is one of the themes in asthma drug-related research works.

### 4.4 Strengths and limitations

The two important limitations of the current study should be pointed out. First, we only analyzed the studies indexed in the WOSCC database and only included English publications. Second, the quality of the included literature was uneven, which may lead to some degree of deviation in the analysis. Third, the Matthew effect, which might influence the results of bibliometric analysis, was not considered (Jiang et al., 2021).

## 5 Conclusion

This study demonstrates the global research hotspots and trends of the research works on drugs for patients with asthma/severe asthma. It can help scholars to quickly understand the current status and hotspots of research in this field.

## Data Availability

The original contributions presented in the study are included in the article/Supplementary Material; further inquiries can be directed to the corresponding authors.

## Supplementary material

SUPPLEMENTARY FIGURE S1 Flow chart of the search strategy about asthma drug-related research.

SUPPLEMENTARY FIGURE S2 Annual publication number in the field of asthma and severe asthma drug-related research.

SUPPLEMENTARY FIGURE S3 Annual publications of top five countries from 1982 to 2021.

SUPPLEMENTARY FIGURE S4 Annual publications of top five institutions (non-pharmaceutical companies) from 1982 to 2021.

SUPPLEMENTARY FIGURE S5 Top 25 cited journals with strongest citation bursts in asthma drug-related studies. Note: OA: open-access journal.

SUPPLEMENTARY FIGURE S6 Annual number of asthma drug-related studies of each drug from 1982 to 2021. Notes: **(A)**: asthma drugs and **(B)**: asthma drug categories.

SUPPLEMENTARY FIGURE S7 Annual number of severe asthma drug-related studies of each drug.
