# Supplementary figures and images for: A bibliometric and scientific knowledge map study of the drug therapies for asthma-related study from 1982 to 2021
Source: Front Pharmacol. 2022 Oct 3;13:916871. doi: 10.3389/fphar.2022.916871 (PMC9574019; doi:10.3389/fphar.2022.916871)

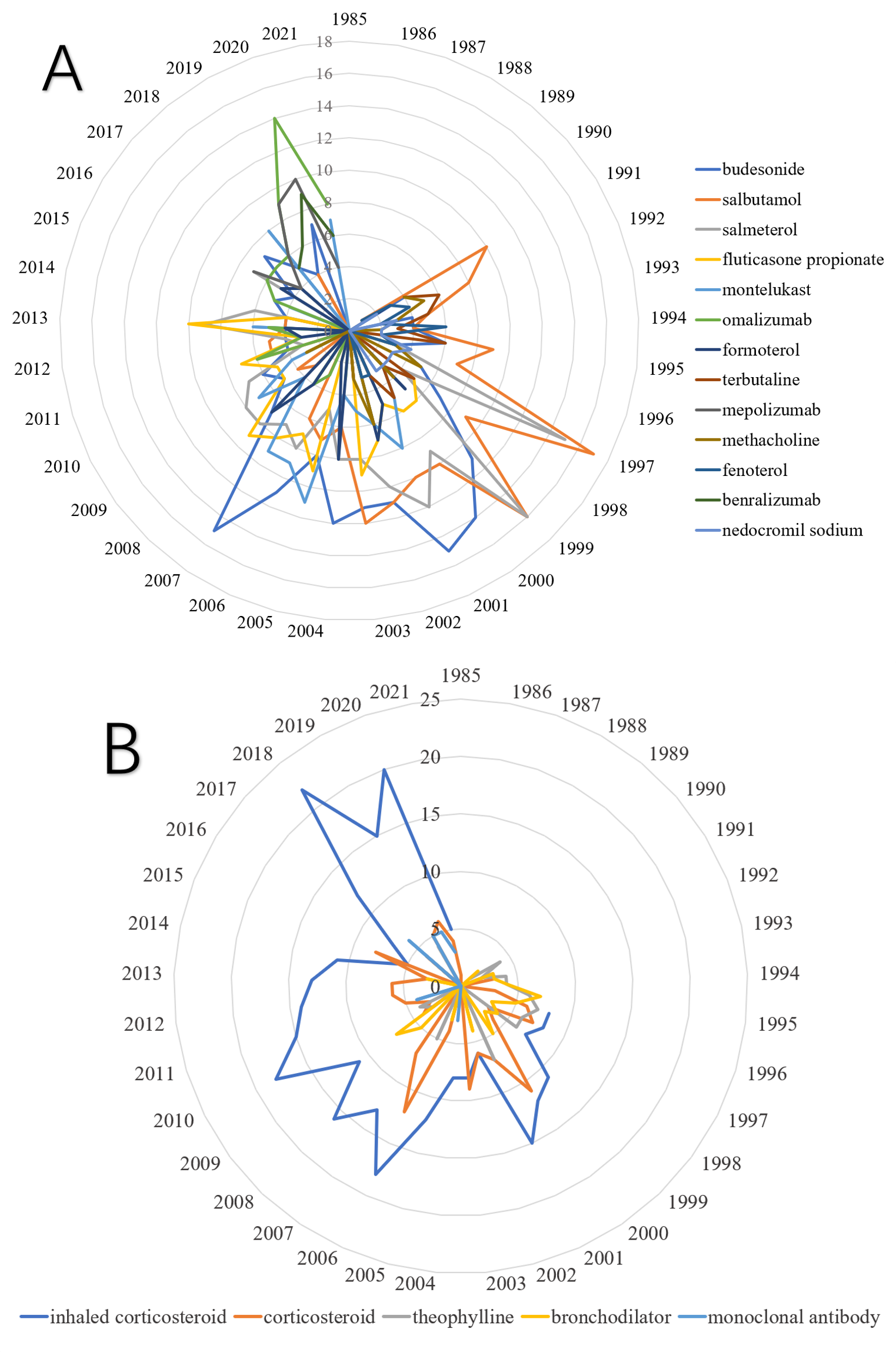

Supplement: Supplementary file 1 [file Image6.tif]

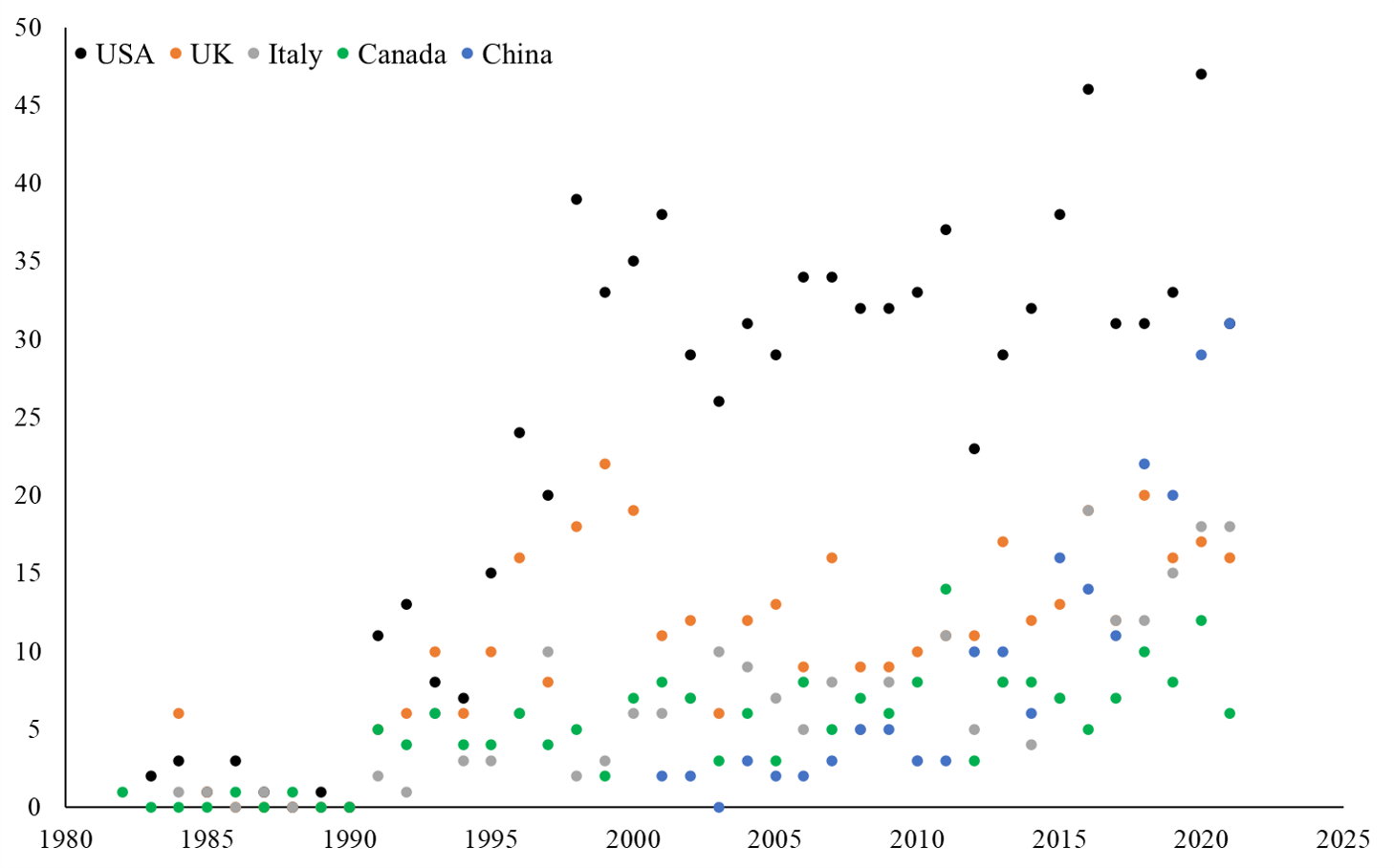

Supplement: Supplementary file 3 [file Image3.tif]

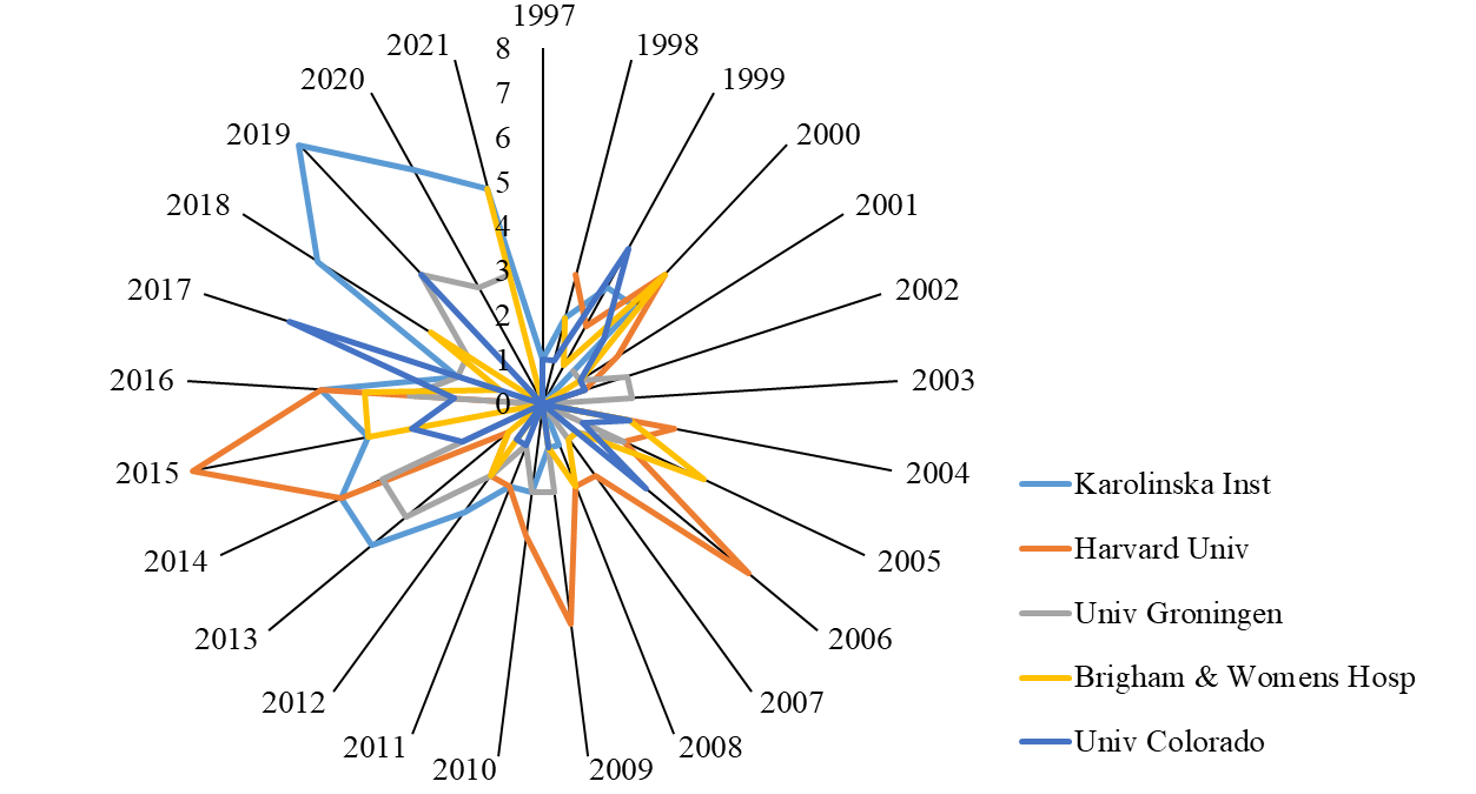

Supplement: Supplementary file 4 [file Image4.tif]

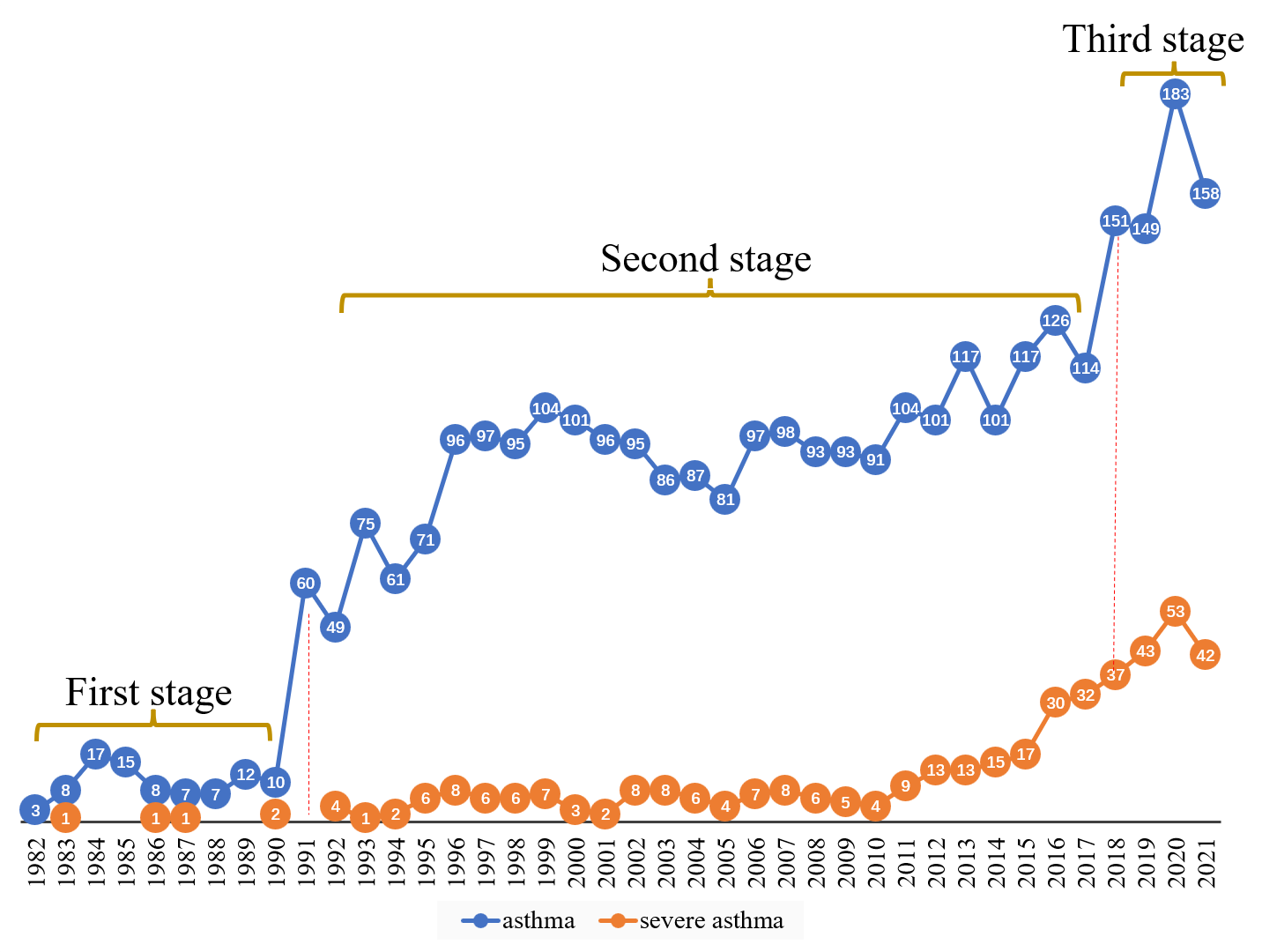

Supplement: Supplementary file 5 [file Image2.tif]

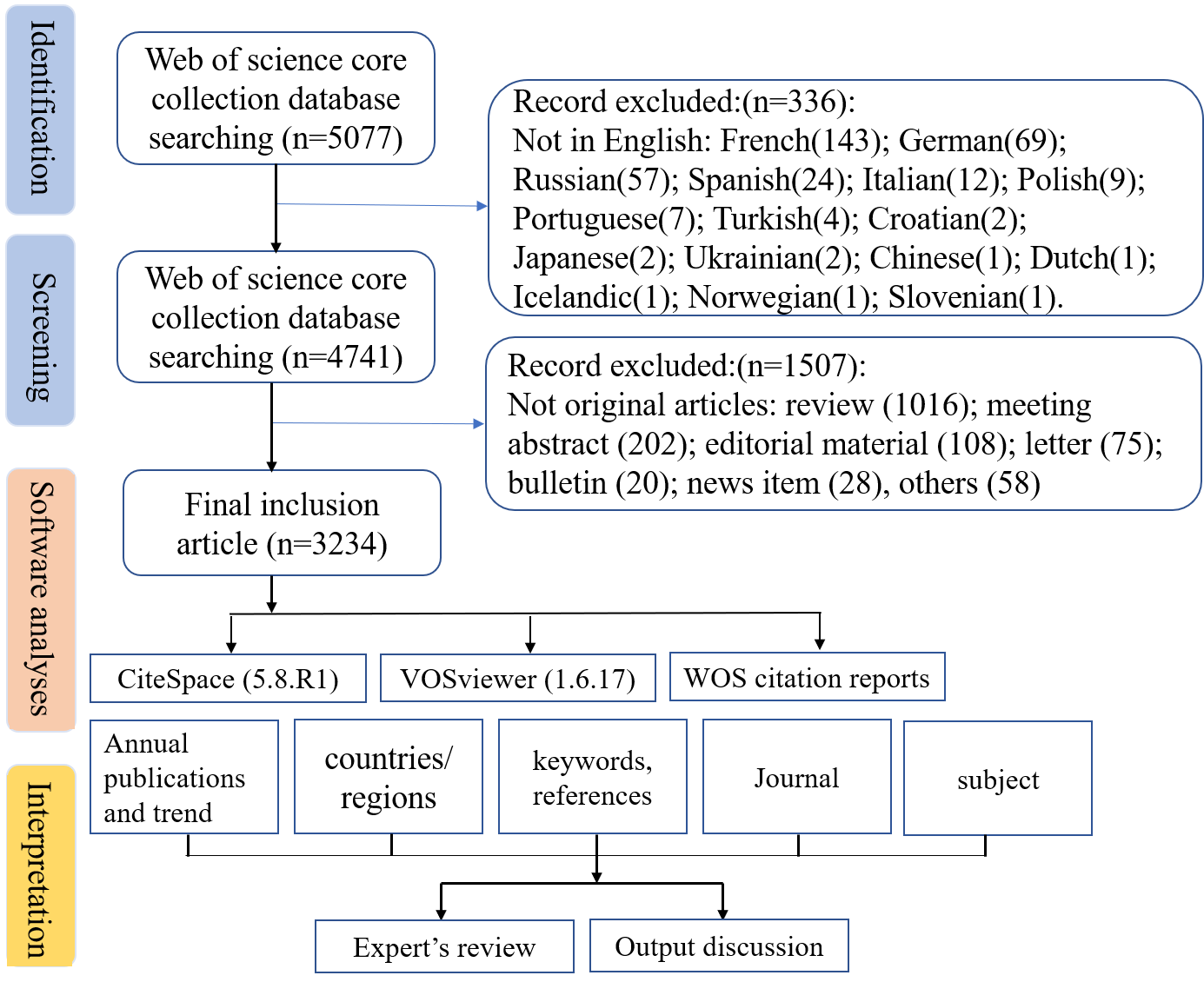

Supplement: Supplementary file 6 [file Image1.tif]

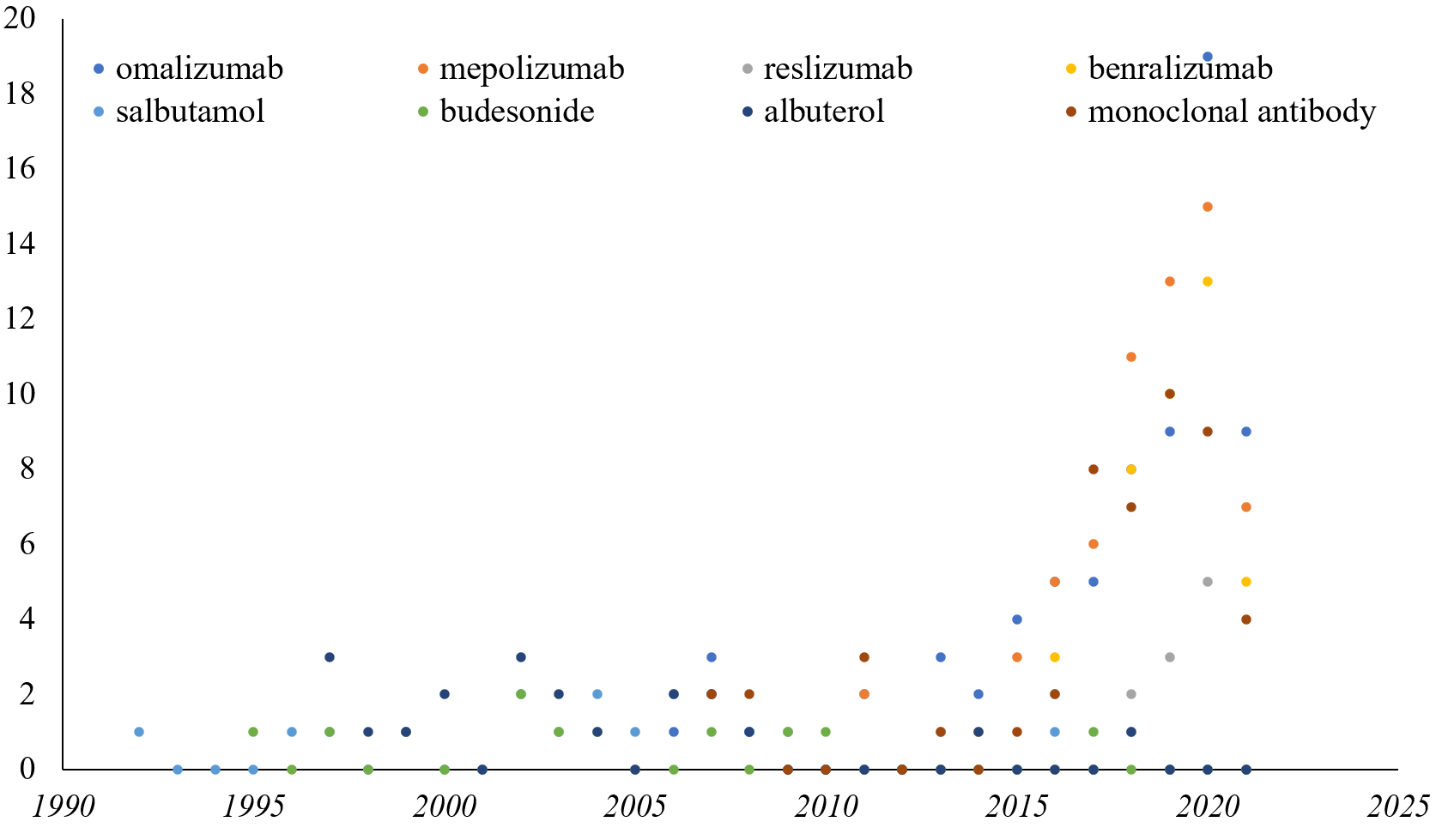

Supplement: Supplementary file 7 [file Image7.tif]

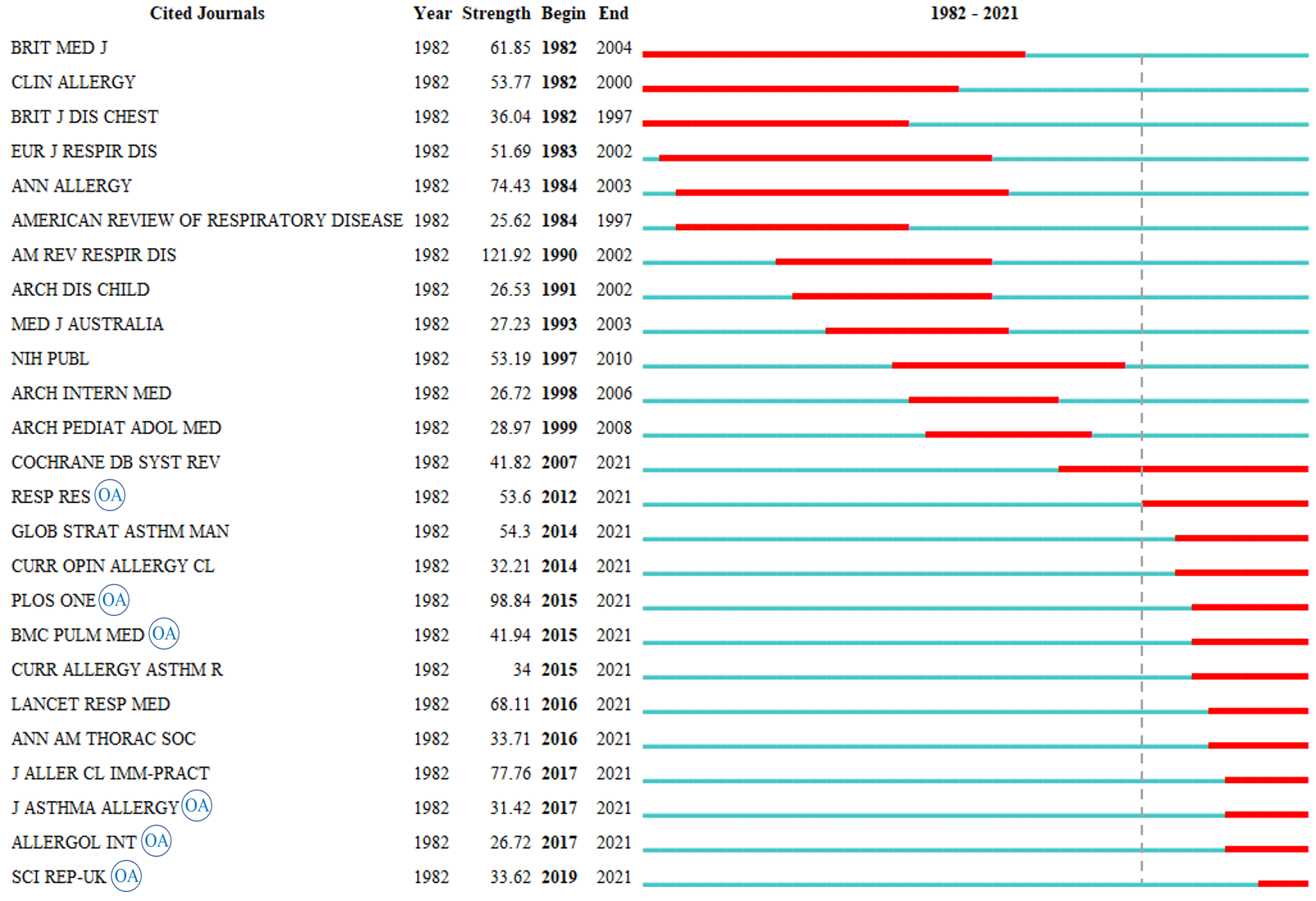

Supplement: Supplementary file 9 [file Image5.tif]
