# Supplementary material for: A bibliometric and scientific knowledge map study of the drug therapies for asthma-related study from 1982 to 2021
Source: Front Pharmacol. 2022 Oct 3;13:916871. doi: 10.3389/fphar.2022.916871 (PMC9574019; doi:10.3389/fphar.2022.916871)
Supplement: Supplementary file 2 [file Table1.docx]

Supplements Table 1. Distribution of publications from top 20 institutions

| **Rank** | **institutions** | **count** | **centrality** | **year** |
| --- | --- | --- | --- | --- |
| 1 | GSK (UK) | 84 | 0.15 | 1997 |
| 2 | Karolinska Inst (Sweden) | 67 | 0.04 | 1997 |
| 3 | AstraZeneca (UK) | 62 | 0.11 | 2000 |
| 4 | Harvard Univ (USA) | 58 | 0.07 | 1998 |
| 5 | Univ Groningen (Netherland) | 42 | 0.06 | 1999 |
| 6 | Brigham & Womens Hosp (USA) | 39 | 0.05 | 1998 |
| 7 | Univ Colorado (USA) | 36 | 0.01 | 1997 |
| 8 | Univ Calif San Francisco (USA) | 35 | 0.03 | 1998 |
| 9 | McMaster Univ (Canada) | 31 | 0.03 | 1998 |
| 10 | McGill Univ (Canada) | 29 | 0.02 | 1998 |
| 11 | Univ Hosp (N/A) | 29 | 0.03 | 1998 |
| 12 | Univ Wisconsin (USA) | 28 | 0.04 | 1998 |
| 13 | Univ Aberdeen (UK) | 27 | 0.02 | 2000 |
| 14 | Univ Toronto (Canada) | 27 | 0.01 | 2000 |
| 15 | Karolinska Univ Hosp (Sweden) | 25 | 0 | 2010 |
| 16 | Univ Genoa (Italy) | 25 | 0.01 | 2001 |
| 17 | Natl Jewish Med & Res Ctr (USA) | 25 | 0.02 | 1997 |
| 18 | Univ British Columbia (Canada) | 25 | 0.02 | 2001 |
| 19 | Univ Cape Town (South Africa) | 24 | 0.03 | 1998 |
| 20 | Univ London Imperial Coll Sci Technol & Med (UK) | 23 | 0.02 | 1998 |
