# Supplementary material for: A bibliometric and scientific knowledge map study of the drug therapies for asthma-related study from 1982 to 2021
Source: Front Pharmacol. 2022 Oct 3;13:916871. doi: 10.3389/fphar.2022.916871 (PMC9574019; doi:10.3389/fphar.2022.916871)
Supplement: Supplementary file 8 [file Table2.docx]

Supplementary Table 2. Top 20 journals with most articles and most citations

| **Rank** | **Journals with most articles (WOSCC)** | **count** | **Journals with most citations (Citespace)** | **count** | **centrality** |
| --- | --- | --- | --- | --- | --- |
| 1 | J Asthma | 161 | J Allergy Clin Immun | 2199 | 0.03 |
| 2 | J Allergy Clin Immun | 157 | Eur Respir J | 1709 | 0.07 |
| 3 | Resp Med | 103 | Am J Resp Crit Care | 1686 | 0.02 |
| 4 | Eur Respir J | 81 | Thorax | 1535 | 0.05 |
| 5 | Chest | 77 | Chest | 1454 | 0.03 |
| 6 | Ann Allerg Asthma Im | 75 | New Engl J Med | 1411 | 0.02 |
| 7 | Thorax | 66 | Lancet | 1212 | 0.03 |
| 8 | Allergy | 56 | Resp Med | 1033 | 0.03 |
| 9 | Am J Resp Crit Care | 49 | Allergy | 1007 | 0.08 |
| 10 | Clin Exp Allergy | 49 | Am Rev Respir Dis | 956 | 0.04 |
| 11 | Pediatr Pulm | 44 | J Asthma | 848 | 0.18 |
| 12 | Pulm Pharmacol Ther | 39 | Clin Exp Allergy | 847 | 0.03 |
| 13 | Allergy Asthma Proc | 35 | Ann Allerg Asthma Im | 767 | 0.02 |
| 14 | Int Arch Allergy Imm | 33 | Brit Med J | 625 | 0.02 |
| 15 | Plos One | 28 | JAMA-J Am Med Assoc | 562 | 0.05 |
| 16 | Pediatrics | 27 | Pediatrics | 524 | 0.03 |
| 17 | Pediat Allerg Imm-uk | 26 | J Immunol | 450 | 0.07 |
| 18 | Curr Med Res Opin | 23 | J Clin Invest | 410 | 0.06 |
| 19 | J Allergy Clin Immun | 23 | Bmj-brit Med J | 382 | 0.04 |
| 20 | Pharmacoepidem Dr S | 23 | Ann Intern Med | 372 | 0.06 |
